# Author Correction: Genomic analysis finds no evidence of canonical eukaryotic DNA processing complexes in a free-living protist

**DOI:** 10.1038/s41467-021-27605-w

**Published:** 2021-12-16

**Authors:** Dayana E. Salas-Leiva, Eelco C. Tromer, Bruce A. Curtis, Jon Jerlström-Hultqvist, Martin Kolisko, Zhenzhen Yi, Joan S. Salas-Leiva, Lucie Gallot-Lavallée, Shelby K. Williams, Geert J. P. L. Kops, John M. Archibald, Alastair G. B. Simpson, Andrew J. Roger

**Affiliations:** 1 Institute for Comparative Genomics (ICG), Department of Biochemistry and Molecular Biology, Dalhousie University, Halifax, NS B3H 4R2 Canada; 2 Department of Biochemistry, University of Cambridge, Cambridge, United Kingdom; 3 Groningen Biomolecular Sciences and Biotechnology Institute, University of Groningen, Groningen, The Netherlands; 4 Institute of Parasitology, Biology Centre, Czech Academy of Science, České Budějovice, Czech Republic; 5 Guangzhou Key Laboratory of Subtropical Biodiversity and Biomonitoring, School of Life Science, South China Normal University, Guangzhou, 510631 China; 6 CONACyT-Centro de Investigación en Materiales Avanzados, Departamento de medio ambiente y energía, Miguel de Cervantes 120, Complejo Industrial Chihuahua, 31136 Chihuahua, Chihuahua México; 7 Oncode Institute, Hubrecht Institute – KNAW (Royal Netherlands Academy of Arts and Sciences) and University Medical Centre Utrecht, Utrecht, The Netherlands; 8 Institute for Comparative Genomics (ICG), Department of Biology, Dalhousie University, Halifax, NS B3H 4R2 Canada

**Keywords:** Evolution, Microbiology

Correction to: *Nature Communications* 10.1038/s41467-021-26077-2; published online 14 October 2021.

The original version of this Article contained an error in Fig. 1 panel b, which presented a left-handed helix instead of a right-handed helix conformation. Furthermore the labels for the 5' and 3' DNA ends and arrows to indicate the progression of DNA synthesis were missing.

The correct version of Fig. 1 is :
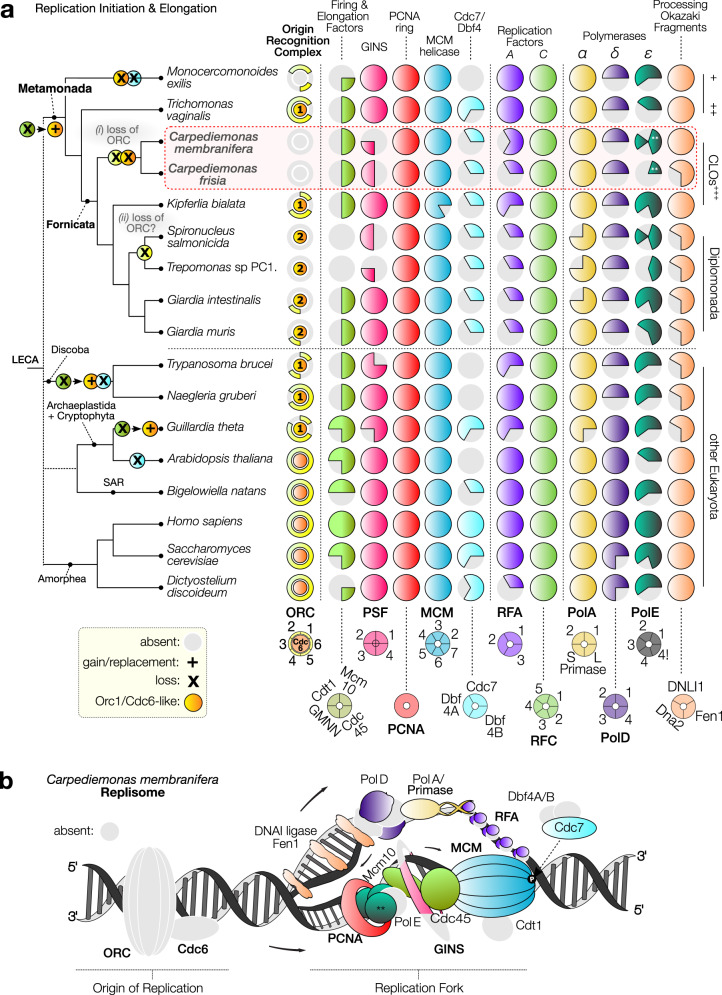


which replaces the previous incorrect version:
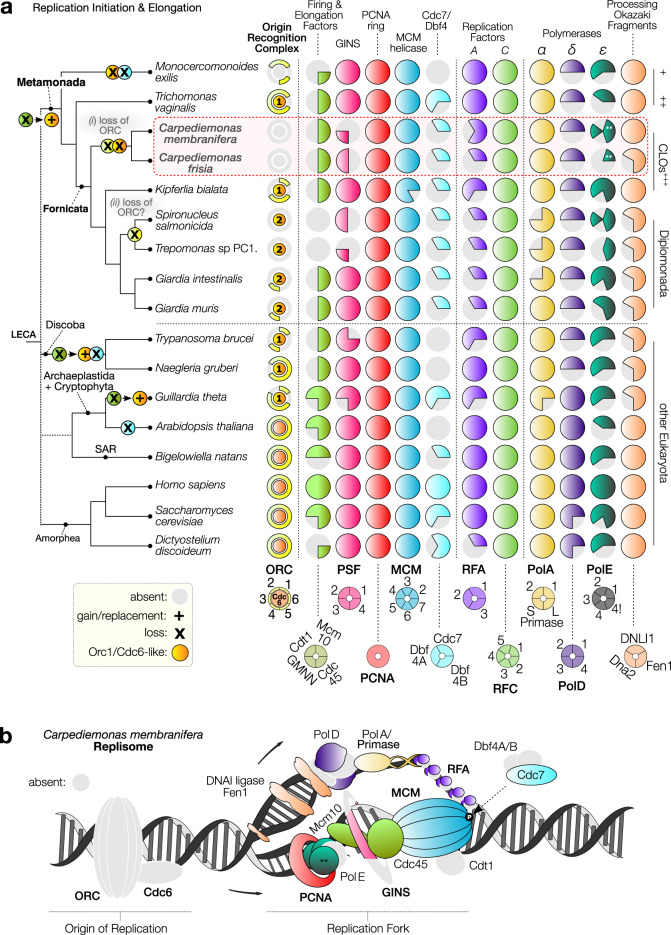


The original version of this Article also contained an error in Fig. 4b and c, in which the strands of the Holliday junction were switched and Mre11-Rad50 was implicated as a 5’-3’ exonuclease.

The correct version of Fig. 4 is :
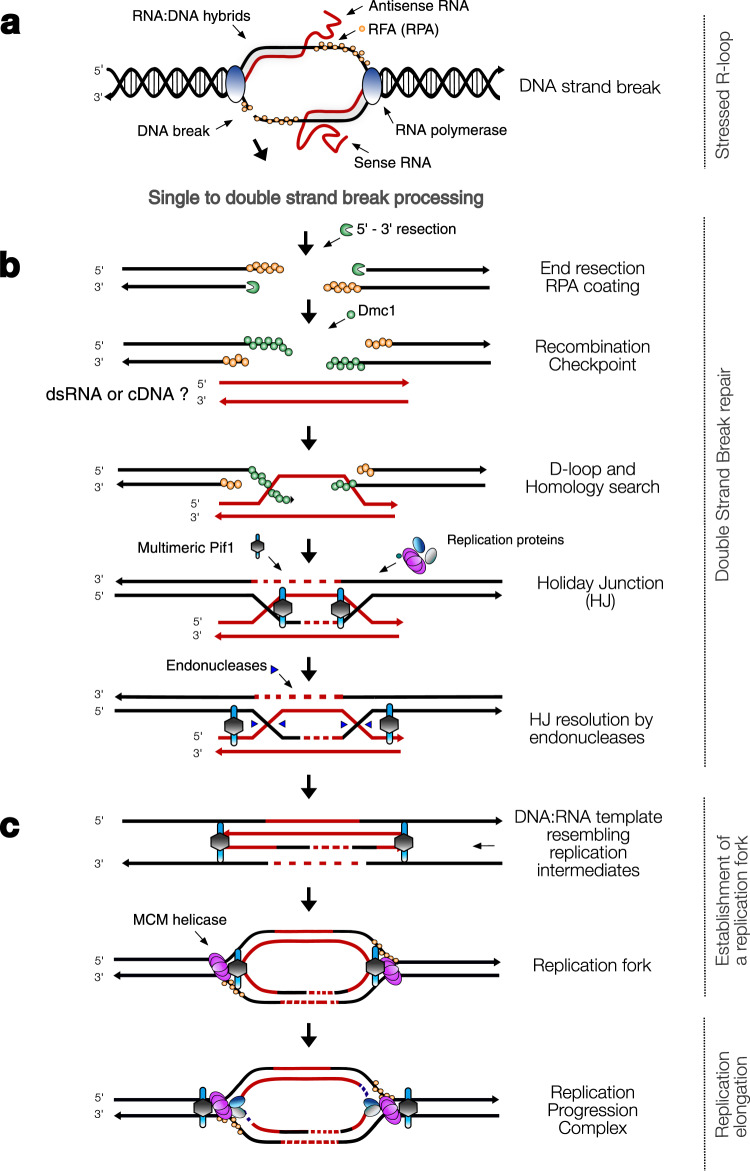


which replaces the previous incorrect version:
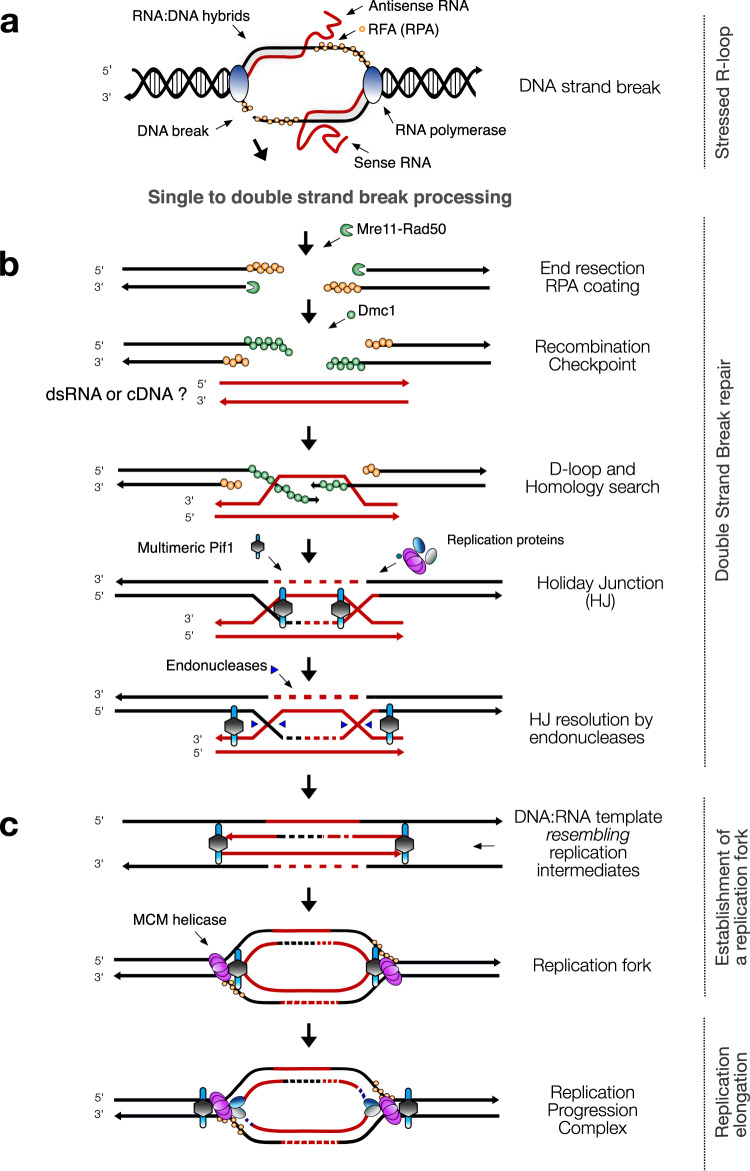


The legend for Fig. 4 incorrectly read ‘**b** Once the damage is processed into a DSB, end resection by Mre11/Rad50 creates a 3′ overhang and the strands are coated with replication protein A (RPA), while resected ends are coated with the recombinase Dmc1. ’ The correct version states ‘**b** Once the damage is processed into a DSB, end resection creates an overhang, and the strands are coated with replication protein A (RPA), and the recombinase Dmc1.’

The legend also incorrectly stated ‘Dark blue fragments on 3′ ends of the bottom figure represent Okazaki fragments.’. In the corrected version, ‘3’’ has been removed.

This has been corrected in both the PDF and HTML versions of the Article.

