# Supplementary material for: Genomic analysis finds no evidence of canonical eukaryotic DNA processing complexes in a free-living protist
Source: Nat Commun. 2021 Oct 14;12:6003. doi: 10.1038/s41467-021-26077-2 (PMC8516963; doi:10.1038/s41467-021-26077-2)
Supplement: Supplementary file 1 — Supplementary Information [file 41467_2021_26077_MOESM1_ESM.pdf]

Supplementary Information for:

Genomic analysis finds no evidence of canonical eukaryotic DNA processing complexes in a free-living protist

Dayana E. Salas-Leiva[1,2*], Eelco C. Tromer[2,3], Bruce A. Curtis[1], Jon Jerlström-Hultqvist[1], Martin Kolisko[4], Zhenzhen Yi[5], Joan S. Salas-Leiva[6], Lucie Gallot-Lavallée[1], Shelby K. Williams[1], Geert J. P. L. Kops[7], John M. Archibald[1], Alastair G. B. Simpson[8] and Andrew J. Roger[1*]

[1] Institute for Comparative Genomics (ICG), Department of Biochemistry and Molecular Biology, Dalhousie University, Halifax, NS, Canada, B3H 4R2

[2] Department of Biochemistry, University of Cambridge, Cambridge, United Kingdom

[3] Groningen Biomolecular Sciences and Biotechnology Institute, University of Groningen, Groningen, Netherlands

[4] Institute of Parasitology Biology Centre, Czech Acad. Sci, České Budějovice, Czech Republic

[5] Guangzhou Key Laboratory of Subtropical Biodiversity and Biomonitoring, School of Life Science, South China Normal University, Guangzhou 510631, China

[6] CONACyT-Centro de Investigación en Materiales Avanzados, Departamento de medio ambiente y energía, Miguel de Cervantes 120, Complejo Industrial Chihuahua, 31136 Chihuahua, Chih., México

[7] Oncode Institute, Hubrecht Institute – KNAW (Royal Netherlands Academy of Arts and Sciences) and University Medical Centre Utrecht, Utrecht, The Netherlands

[8] Institute for Comparative Genomics (ICG), Department of Biology, Dalhousie University, Halifax, NS, Canada, B3H 4R2

[*]corresponding authors: Andrew.Roger@dal.ca and Dayana.Salas@dal.ca

Table of Contents

A. Supplementary methods....3

A1. Culturing and DNA isolation....3

A2. Reads processing and genome assembly....3

A3. Genome size and completeness using BUSCO and a phylogeny-guided approach....5

A4. Taxa selected for comparative genomic analysis....5

A5. Phylogenomic analysis....7

A6. Additional strategies used to search for ORC, Cdc6 ad Ndc80 proteins....7

B. Supplementary results....8

B1. BUSCO completeness....8

B2. Proteins with patchy distribution in metamonads....8

B3. Additional search strategies employed to find missing ORC/Cdc6 and Ndc80 proteins....11

B4. DNA replication streamlining in nucleomorphs....12

B5. Acquisition of Endonuclease IV, RarA and RNAse H1 by lateral gene transfer....13

C. Supplementary discussion....14

C1. BUSCO incompleteness....14

D. Supplementary figures....15

E. Supplementary references....30

A. Supplementary methods

A1. Culturing and DNA isolation

Sequencing of *C. membranifera* BICM strain was done with Illumina short paired-end and long MinION read technologies. The Illumina sequencing employed DNA from a monoxenic culture grown in 50 ml Falcon tubes in F/2 media enriched with the bacterium *Shewanella frigidimarina* as food. DNA was isolated from a total of two litres of culture using a salt extraction protocol followed by CsCl gradient centrifugation. RNA was also extracted from these cultures using TRIzol (Invitrogen, USA), following the manufacturer's instructions. For MinION sequencing, *C. membranifera* was grown in sterile filtered 50% natural sea water media with 3% LB with either *Shewanella sp* or *Vibrio sp.* isolate JH43 as food. Cell cultures were harvested at peak density by centrifugation at 500×*g*, 8 min, 20 ºC. The cells were resuspended in sterile-filtered spent growth media (SFSGM) and centrifuged again at 500×*g*, 8 min, 20 ºC. The cell pellets were resuspended in 1.5 mL SFSGM, layered on top of 9 mL Histopaque®-1077 (Sigma-Aldrich) and centrifuged at 2000×*g*, 20 min, 20 ºC. The protists were recovered from the media:Histopaque interface by pipetting, diluted in 10 volumes of SFSGM and centrifuged 500×*g*, 8 min, 20 ºC. High molecular weight DNA was extracted using MagAttract HMW DNA Kit (Qiagen, Cat No. 67563), purified with GenomicTip 20/G (Qiagen, Cat No. 10223), and resuspended in 5 mM Tris-HCl (pH 8.5).

A2. Reads processing and genome assembly

Long reads were base-called and trimmed with Albacore v2.3.3 (www.nanoporetech.com) and Porechop v0.2.3[1], respectively. ABruijn v1.0[2] with default parameters and max genome size of 30Mb produced an assembly that was polished with Nanopolish v0.10.1[3]. The latter was iteratively error-corrected with the genomic paired-end Illumina reads using the stand-alone tool 'unicycler_polish' from Unicycler v0.4.4[4]. The tool uses short reads to do iterative correction on a provided assembly by

wrapping on Pilon[5] and Bowtie2[6] programs. These two programs are usually used in combination for polishing/error-correction to correct any sequences from long read technologies, and we chose to use them as implemented in 'unicycler_polish' because: 1) *C. membranifera*'s genome is small (the code is not optimized for large assemblies) and predicted as haploid (personal communication with R. Wick[4] suggested that its use on a haploid eukaryotic genome should be fine as problems would be expected due to unphased assemblies or ploidy levels), and 2) the stand-alone tool uses an iterative approach evaluating whether there were improvements or not in each round of corrections; hence, it determines if additional correction rounds are needed. We note that we did not use the ALE-guided correction of larger variants output (one of the outputs), instead, we used the output produced in round 8 (Supplementary Table 1). The identification and removal of prokaryotic contigs was assisted by BLASTn[7] v2.7.1 searches against the nt database with the following cut-offs: percentage identity ≥ 40%, query coverage ≥ 60% and e-value of 10^{-3}. Read-depth coverage at each position of the genomic scaffolds were obtained with SAMtools v1.11[8] and mosdepth v0.2.5[9].

Supplementary Table 1 Corrections done by each round with Unicycler_polishing tool

Round	Variants applied after the round	Homopolymer corrections	Insertions	Deletions	Substitutions
1	16804	12098	924	872	2910
2	543	143	60	74	266
3	191	44	29	20	98
4	101	19	11	6	65
5	85	12	4	5	64
6	55	6	4	6	39
7	47	6	2	2	37
8	10	4	0	2	4

A3. Genome size and completeness using BUSCO and a phylogeny-guided approach

The BUSCO approach[10] was prone to false negative predictions with our dataset because of the high divergence of metamonad homologs. Therefore, the completeness of the BUSCO set was re-assessed with a phylogeny-guided search. For this, we eliminated 31 proteins associated with mitochondria or mitochondrion- related organelles (MROs) as Metamonada have reduced or no MROs[11], and employed taxa-enriched Hidden Markov Model (HMM) searches to account for divergence between the remaining 272 proteins and the studied taxa. In brief: BLASTp was carried out using the 272 BUSCO proteins as queries for finding their orthologues in a local version of the PANTHER 14.0 database[12] to enable the identification of the most likely Panther subfamily HMM and its annotation. Then, each corresponding subfamily HMM was searched for in the predicted proteomes with an e-value cut-off of $1x10^{-1}$ with HMMER v3.1b2[13]. In cases where these searches did not produce any result, a broader search was run using the HMM of the Panther family with $1x10^{-3}$ as e-value cut-off. Five best hits for each search were retrieved from each proteome, aligned to the corresponding Panther subfamily or family sequences with MAFFT v7.310[14] and phylogenetic reconstructions were carried out using IQ-TREE v1.6.5[15] under the LG+C60+F+Γ model with ultrafast bootstrapping (1000 replicates). Protein domain architectures were visualized by mapping the respective Pfam v33.1 accessions onto trees using ETE tools v3.1.1[16].

A4. Taxa selected for comparative genomic analysis

Our analyses included the publicly available genomes and predicted proteomes of *Trichomonas vaginalis* G3[17] (Parabasalia, www.trichdb.org), *Monocercomonoides exilis*[18] (Preaxostyla, www.protistologie.cz/hampllab), the free-living fornicates *Carpediemonas frisia*[19] (*i.e.*, metagenomic bin and predicted proteome), *Carpediemonas membranifera* (reported here) and *Kipferlia bialata*[20], plus the parasitic diplomonad fornicates: *Giardia intestinalis* Assemblages A[21] and B[22], *Giardia*

muris[23] (Note: a higher quality assembly for *G. intestinalis* A was recently published and contains 938 genes less than the assembly we used, but has on average longer genes and smaller intergenic regions[24]. Our analyses only considered the assembly reported in[21]), *Spironucleus salmonicida* ATCC50377[25] (www.giardiadb.org) and *Trepomonas* sp. PC1[26] −the latter was only available as a transcriptome. We also included a set of genomes that are broadly representative of eukaryote diversity, such as *Homo sapiens* GRCh38[27], *Saccharomyces cerevisiae* S288C 2010 (https://www.yeastgenome.org/), *Arabidopsis thaliana* TAIR10[28] (https://www.arabidopsis.org/), *Dictyostelium discoideum* AX4[29], *Trypanosoma brucei* TREU927[30] (www.uniprot.org), *Naegleria gruberi* NEG-M[31] (www.ncbi.nlm.nih.gov), *Guillardia theta* and *Bigelowiella natans*[32] (www.genome.jgi.doe.gov/portal/).

Additional analyzed genomes were those of the microsporidia *Encephalitozoon intestinalis* ATCC 50506[33] (ASM14646v1), *E. cuniculi* GB-M1[34] (ASM9122v2) and *Trachipleistophora hominis*[35] (ASM31613v1), the yeasts *Hanseniaspora guilliermondii*[36](ASM491977v1), *Hanseniaspora opuntiae*[37] (ASM174979v1), *Hanseniaspora osmophila*[37] (ASM174704v1), *Hanseniaspora uvarum*[37] (ASM174705v1) and *Hanseniaspora valbyensis* NRRL Y-1626[38] (GCA_001664025.1), the metamonad *Tritrichomonas foetus*[39] (ASM183968v1), the nucleomorphs of *Hemiselmis andersenii*[40] (ASM1864v1), *Cryptomonas paramecium*[41] (ASM19445v1), *Chroomonas mesostigsmatica*[42] (ASM28609v1), *Guillardia theta*[43](ASM297v1), *Lotharella vacuolata*[44] (AB996599–AB996601), *Amorphochlora amoebiformis*[44] (AB996602–AB996604) and *Bigellowiela natans*[45] (ASM245v1), the corals *Galaxea fascicularis*, *Fungia sp*., *Goniastrea aspera*, *Acropora tenuis* and the coral endosymbionts *Symbiodinium kawagutii* and *Symbiodinium goreaui*[46,47].

A5. Phylogenomic analysis

A previously constructed phylogenomic dataset and pipeline published by Brown et al.[48] was used to obtain alignments of 351 highly conserved protein orthologs from a total of 29 eukaryotic genomes and transcriptomes (for taxa sources see ref[49]). Orthologs from *C. membranifera* and *C. frisia* were added to that dataset, which was further sub-selected to avoid those with known deep-paralogy, and to maximize alignment site coverage amongst taxa of interest. This resulted in 181 highly conserved genes, encompassing 19 metamonads and other outgroup 12 eukaryotes, that were aligned and then concatenated. The alignment was done with MAFFT v7.310[14] (mafft-linsi option) and trimmed with BMGE v1.0[50] with default parameters. Initially, a guide tree was estimated by maximum likelihood using IQ-TREE[15] with the LG+C60+F+Γ model and 1000 ultrafast bootstraps. This was used to estimate the PMSF profiles for tree inference under the LG+PMSF(C60)+F+ Γ model for 100 nonparametric bootstraps, approximate likelihood ratio tests and aBayes support tests.

A6. Additional strategies used to search for ORC, Cdc6 ad Ndc80 proteins

Strategies included enriched HMMs as mentioned in the main text and HMMs for individual Pfam domains with e-value thresholds of 1×10^{-3}. 1) Metamonad-specific HMMs were built as described for kinetochore proteins − containing the newly found hits plus orthologs from additional publicly available metamonad proteomes or transcriptomes[11,39], 2) we applied the eggNOG 4.5 profiles COG1474, COG5575, KOG2538, KOG2228, KOG2543, KOG4557, KOG4762, KOG0995, KOG4438, KOG4657 and 2S26V which encompass 2774, 495, 452, 466, 464, 225, 383 , 504, 515, 403 and 84 taxa, respectively, and 3) the Pfam v33.1 HMMs: PF09079 (Cdc6_C), PF17872 (AAA_lid_10), PF00004 (AAA+), PF13401 (AAA_22), PF13191 (AAA_16), PF01426 (BAH), PF04084 (Orc2), PF07034 (Orc3), PF18137 (ORC_WH_C) , PF14629 (Orc4_C), PF14630 (Orc5_C), PF05460 (Orc6), PF03801 (Ndc80_HEC), PF03800 (Nuf2), PF08234 (Spindle_Spc25)

and PF08286 (Spc24). For Ncd80, Nuf2, Spc24 and Spc25 we also applied the HMMs models published in[51].

B. Supplementary results

B1. BUSCO completeness

A subset of 272 BUSCO proteins from the odb9 database was used for a phylogeny-guided search for divergent orthologs. This revealed that: *i*) 27 out of 272 BUSCO (9.9%) proteins are absent in all metamonads, *ii*) only 101 (~41%) of the remaining 245 proteins were shared by all metamonad proteomes, and *iii*) up to 38% are absent in all Fornicata. Metamonad genomes only contained 60% to 91% of the BUSCO proteins (Table 1, Supplementary Data 1, note that the BUSCO presence-absence patterns of the transcriptomic data from *Trepomonas* sp. PC1 are consistent with those of the remaining diplomonads). These analyses demonstrate that the Metamonada have secondarily lost a relatively large number of highly conserved eukaryotic proteins and, therefore, BUSCO analysis cannot be used on its own to evaluate metamonad genome completeness.

B2. Proteins with patchy distribution in metamonads

The replisome proteins Cdt1, Mcm10, Cdc45, GINS subunits 1 and 3, Dbf4 (A and B), subunits 2 and 3 of RFA, and subunits 3 and 4 of polymerase δ and ε vary in their presence/absence distribution pattern across non-metamonad eukaryotes suggesting that some of these are apparently not essential, but their loss could lead to some degree of function impairment. In fact, polymerase δ and ε subunits 3 and 4 are typically considered accessory[52,53], and the same designation may apply to proteins such as Cdt1, Mcm10 and Dbf4, which rarely have been reported outside of Viridiplantae and Opisthokonta (see taxonomy reports for KOG4762 (Cdt1), KOG3056 (Mcm10), COG5067 (Dbf4) at http://eggnog5.embl.de/). However, there is experimental evidence supporting serious function

impairment when the recruitment of some proteins is compromised (*e.g.*, GINS, Cdc45, RFA)[54,55]. Therefore, we suspect that the absence of some of these subunits, although only detected in a few non-metamonad taxa in our study, may be indicative of unstable replisomes in the organisms lacking them. Some of these absence patterns were also observed in metamonads, for example, subunits of polymerases δ and ε are missing, consistent with their 'accessory' designation. Although, it is notable that the degree of depletion in subunits of GINS, RFA, ORC is far more pronounced in Fornicata than in the Parabasalida and Preaxostyla. Experimental investigations are needed to elucidate how the replisomes of these metamonads function – specially in fornicates – with these greatly reduced or absent complexes.

In terms of the BER and NER pathways, many proteins are not found in any metamonads (*e.g.*, Pol B, Ligase III, OGG1, XPC, XPA) (Supplementary Data 2) and therefore could have been lost prior to the last common ancestor of the group. The absence of Pol B from the BER pathway is intriguing and suggests that a different polymerase should have taken up its task, especially because only long-patch BER pathway would be enabled in metamonads. The patterns of NER proteins in metamonads, particularly *K. bialata* and diplomonads, indicate that these are likely to be sensitive to UV exposure (only the diplomonad *G. intestinalis* has been studied in this regard[22,56,57]). In the MMR pathway, we found a near complete set of proteins from the MutL family in metamonads (*i.e.*, Mlh1, Mlh2 and Mlh3) with orthologs that are highly divergent but conserve the domain architecture of the protein family. In contrast, the MutS protein family has several missing orthologs with only Msh2 and Msh6 (Msh6-like in diplomonads) shared by all metamonads, and Msh4 and Msh5 only absent in diplomonads (note that Msh4 and Msh5 do not participate in MMR but are implicated in meiosis[58]). The loss of Msh3 in *T. vaginalis*, *Carpediemonas* species and diplomonads suggests that these taxa are only able to repair base-base and small insertion/deletion mismatches by using Msh2-Msh6 or

Msh2-Msh6-like (MutSα) but not larger insertion/deletion mismatches as the heterodimer Msh2-Msh3 (MutSβ) could not be formed[59].

In terms of damage signaling, we speculate that, due to the consistent patchiness of the checkpoint proteins Chk1 and Chk2 over all eukaryotes we examined, other kinases probably have taken over their roles in multiple separate lineages. The remaining damage sensing proteins and recombinases in *T. vaginalis* and *M. exilis* indicate that these taxa likely have slightly modified complexes that would be expected to conserve their function (Supplementary Data 2, Supplementary Fig. 5). For example, whereas the complex BCDX2 (Rad51B-Rad51C-Rad51D-Xrcc2), that is responsible for facilitating the assembly and stability of the Rad51 filament, is completely absent in fornicates, a modified version occurs in *M. exilis* (*i.e.*, Rad51B-Rad51C-Xrcc2) and a different one in *T. vaginalis* (*i.e.*, Rad51C-Rad51D-Xrcc2).

Mitosis and meiosis are very distinctive processes that, besides using the recombination machinery and checkpoint controls previously described, use multiple members from the SMC and Rad21 families, among others. Metamonads have all Condensin I and II, Cohesin, and Smc5-Smc6 complexes for chromosome handling. The number of homologs for the Rad21 family, part of the Cohesin complex, varies from fully absent in diplomonads to four paralogs in *M. exilis*. Notably, these proteins are very divergent in *M. exilis, K. bialata* and *C. membranifera,* forming a new Rad21 clade in this family.

It is noteworthy that our findings in all the studied systems provide additional evidence that *M. exilis*, despite the apparent lack of a mitochondrial compartment, has molecular systems that are more complete than those of other metamonads[60].

B3. Additional search strategies employed to find missing ORC/Cdc6 and Ndc80 proteins

Metamonad-specific HMM retrieved two candidates for Orc1/Cdc6 proteins from *C. frisia* (*i.e.*, Cfrisia_2222, Cfrisia_2845) and one from *C. membranifera* (*i.e.*, J8273_3200), and one Orc4 candidate from each *Carpediemonas* species (*i.e.*, Cfrisia_2559, J8273_7545). Further inspection of these hits showed that only the AAA+ region shared similarity among all of these proteins, which is expected as ORC and Cdc6 proteins belong to the ATPase superfamily. However, based on full protein identity, full profile composition and domain architecture, the proteins retrieved with the Orc1/Cdc6 HMM were confidently annotated as Katanin P60 ATPase-containing subunit A1 (Cfrisia_2222), Replication factor C subunits 1 (J8273_3200) and 5 (Cfrisia_2845), and proteins retrieved with Orc4 HMM were members of the Dynein heavy chain (Cfrisia_2559) and AAA-family ATPase families (J8273_7545). The latter is a 744 aa protein that has a C-terminal region with no sequence similarity or amino acid profile frequencies that resembles a Orc4_C Pfam domain from other metamonads or model eukaryotes. All the additional search strategies yielded false positives in *Carpediemonas* species, as these retrieved AAA-family members lacking sequence similarity to orc proteins, showed completely different protein domain architecture than the expected one and were associated with different functional annotation. When reconstructing the domain architecture of ORC and Cdc6 proteins in metamonads, we noted that Fornicata Orc1/Cdc6-like proteins are remarkably smaller (*i.e.*, 1.5 to 3 times smaller) than Orc1 and Cdc6 from the model organisms and other protists used later in phylogenetic reconstruction (Supplementary Fig. 3a and b, Supplementary Data 2, 5 and 6). In most cases, the small proteins lack protein domains rendering a different domain architecture with respect to their homologs in *S. cerevisiae, H. sapiens, A. thaliana* and *T. vaginalis* (Supplementary Fig. 3a, Supplementary Data 5). For example, Orc1 and Cdc6 paralogs in Fornicata lack BAH, and AAA_lid10 and Cdc6_C domains. Protein alignments show that the conserved areas of these proteins correspond to AAA+ domain that have relatively conserved Walker domains A and

B (except MONOS_13325 from *M. exilis*), with a few proteins lacking the arginine finger motif (R-finger) within the Walker B motif (Supplementary Fig. 3c). The latter may negatively affect ATPase activity of the R-finger-less proteins. In an attempt to establish orthology, metamonad Orc1/Cdc6 candidates were used for phylogenetic reconstruction together with publicly available proteins that have reliable annotations for Orc1 and Cdc6, expected domain architecture and/or with experimental evidence of their functional activity in the replisome. Phylogenetic analysis shows that metamonad proteins form separate clades from the *bona fide* Orc1 and Cdc6 sequences (Supplementary Fig. 3d). One of these separate clades encompasses Orc1-b from *T. brucei* that has been shown to participate during DNA replication despite lacking the typical domain architecture[61].

B4. DNA replication streamlining in nucleomorphs

The loss of ORC/Cdc6 accompanied by the partial retention of MCM, PCNA, Cdc45, RFC, GINS and the homologous recombination (HR) recombinase Rad51 was observed in cryptophyte and chlorarachniophyte nucleomorphs (Supplementary Fig. 4). ORC and Cdc6 were found as single copy genes (except Orc2) in the nuclear genomes of these two groups; their predicted proteins lack obvious signal and targeting peptides which would likely prevent them from participating in a nucleus-coordinated nucleomorph replication. Hence, nucleomorph DNA replication likely occurs by HR without the assistance of ORC/Cdc6 origin-binding, but this replication might nonetheless be regulated at the transcriptional level by the nucleus as shown by[62]. Many of the remaining nuclear-encoded proteins involved in replication are present in more than one gene copy in those taxa, with several of them containing predicted signal and transit peptides (*e.g.*, H2A, Pol D, RFC1 and RFA1)[62,63].

B5. Acquisition of Endonuclease IV, RarA and RNAse H1 by lateral gene transfer

The Endonuclease IV (Apn1 in yeast) and exonuclease III (Exo III) function in the removal of abasic sites in DNA via the BER pathway. Our analyses show that *C. frisia* and *C. membranifera* have Exo III and have a prokaryotic version of Endo IV (Supplementary Fig. 10). Interestingly, none of the parabasalids and *Giardia* spp. have an Endo IV homolog, either eukaryotic or prokaryotic. *S. salmonicida* and *Trepomonas* sp. PC1, by contrast, appear to encode a typical eukaryotic Endo IV.

The RarA (Replication-Associated Recombination protein A, also known as MgsA) protein is ubiquitous in bacteria and eukaryotes (*e.g.*, homologs Msg1 in yeast and WRNIP1 in mammals) and acts in the context of collapsed replication forks[64,65]. *Carpediemonas* possesses a prokaryotic-like version (Supplementary Fig. 11) that lacks the ubiquitin-binding Zn finger N-terminal domain typical of eukaryotic homologs[64]. No canonical eukaryotic RarAs were detected in the remaining metamonads, but it appears that prokaryotic-like RarA proteins in *Giardia*, *S. salmonicida* and *Trepomonas* sp. PC1 were acquired in an independent event from that of *Carpediemonas*.

Both *Carpediemonas* genomes have a eukaryotic RNAse H2, lack eukaryotic RNAse H1 but encode up to two copies of a prokaryotic-like RNAse H1 (Supplementary Fig. 12) which do not have the typical eukaryotic HBD domain[66]. The HBD domain is thought to be responsible for the higher affinity of this protein for DNA/RNA duplexes rather than for dsRNA[67,68]. All prokaryotic-like RNAse H1s in metamonads are highly divergent (Supplementary Fig. 12) and, in the case of *S. salmonicida* RNase H1 proteins, these formed very long branches in all of our preliminary trees, that had to be removed for the final phylogenetic reconstruction. Remarkably, the phylogenetic reconstruction that includes other metamonad proteins suggests that *Giardia*, *Trepomonas* sp. PC1, *T.*

foetus and *T. vaginalis*, also acquired bacterial RNAse H1. *Trepomonas sp*. PC1 and *Giardia* sequences cluster together but the *T. foetus* and *T. vaginalis* enzymes each emerge amidst different bacterial branches, suggesting that they have been acquired independently from the *Carpediemonas* homologs. It should, however, be noted that the support values are overall low, partly due to the fact that these sequences and their relatives are highly divergent from each other, from *Carpediemonas* bacterial-like sequences, and from typical eukaryotic RNaseH1.

C. Supplementary discussion

C1. BUSCO incompleteness

Both eukaryote-wide and protist BUSCO analyses using the BUSCO methods underperformed in our analyses. Despite using a phylogeny-guided search with the Eukaryota database, a more comprehensive database than the protist BUSCO database, a remarkably large number of BUSCO proteins were inconsistently present in Metamonada. This is not surprising, as the clade harbors a very diverse group of taxa with varied lifestyles and many have undergone genome streamlining[20,21,23,25,26], and the BUSCO databases are expected to be more accurate with greater taxonomic proximity to the studied genome[10,69,70]. While it might be tempting to suggest the 101 BUSCO proteins that are shared by all metamonads be used to evaluate genome completion in the clade, the overwhelming evidence of differential genome streamlining strongly indicates that databases should be lineage specific (*e.g.*, *Carpediemonas*, *Giardia*, etc). Hence, our results highlight the need for constructing such databases including proteins that showcase the sequence diversity of the groups and genes that are truly single copy in each of these lineages. Regardless, using only standard BUSCO methods to capture genome completion will still fall short in such assessments as it will fail to evaluate the most difficult-to-assemble regions of the genome[70,71]. For that reason,

combined approaches such as the ones used here provide a more comprehensive global overview of genome completeness.

D. Supplementary figures.

Figures in high resolution are available at Dryad (https://doi.org/10.5061/dryad.wh70rxwnv)

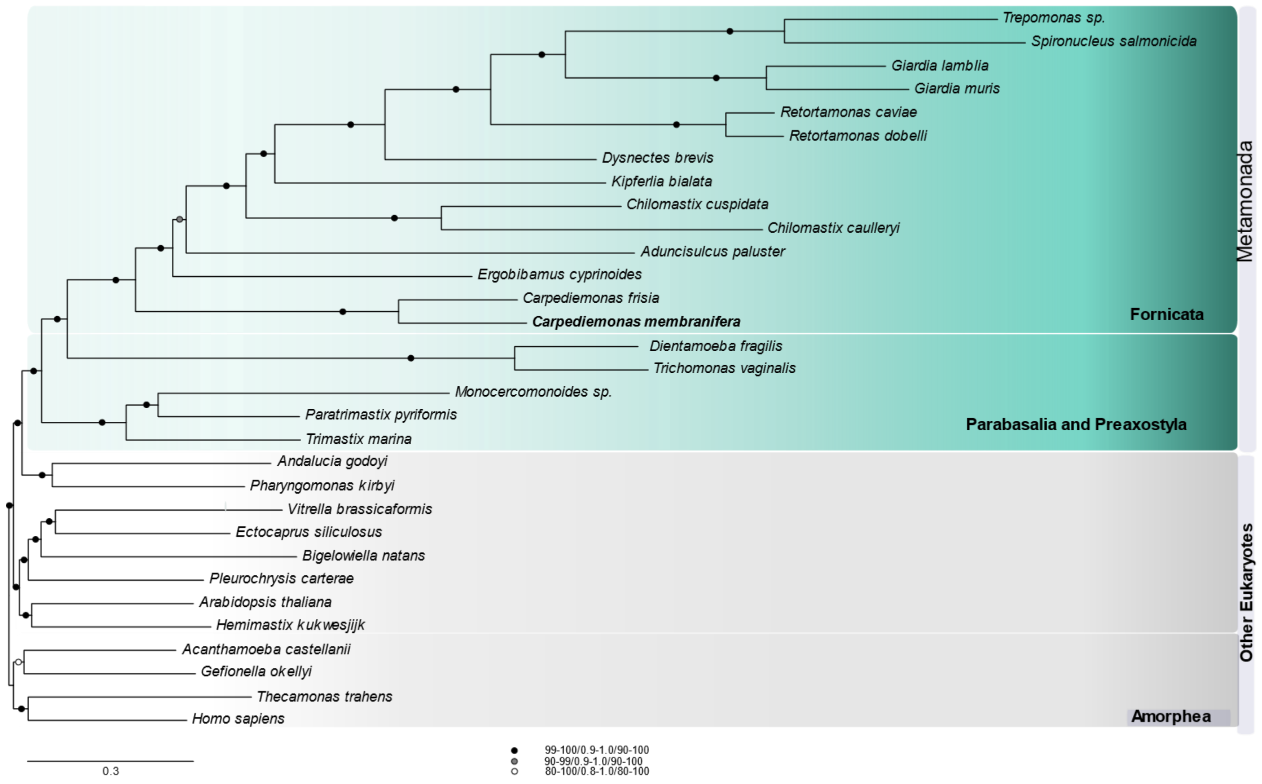

Supplementary Fig. 1 Maximum-likelihood reconstruction of the phylogenetic relationships within the Metamonada clade. An initial reconstruction was carried out in IQ-Tree with the LG+C60+F+Γ model and 1000 ultrafast bootstraps, this was followed by tree inference under LG+PMSF(C60)+F+Γ model using 100 nonparametric bootstraps; alignment length of 181 genes encompassing 48341 sites. Tree rooted on the ancestral branch of Amorphea. Scale bar shows the inferred number of amino acid substitutions per site. Bootstrap values are represented as shaded dots on each branch, and the values are represented in the following order: SH-aLRT support percentage/aBayes/nonparametric bootstrapping.

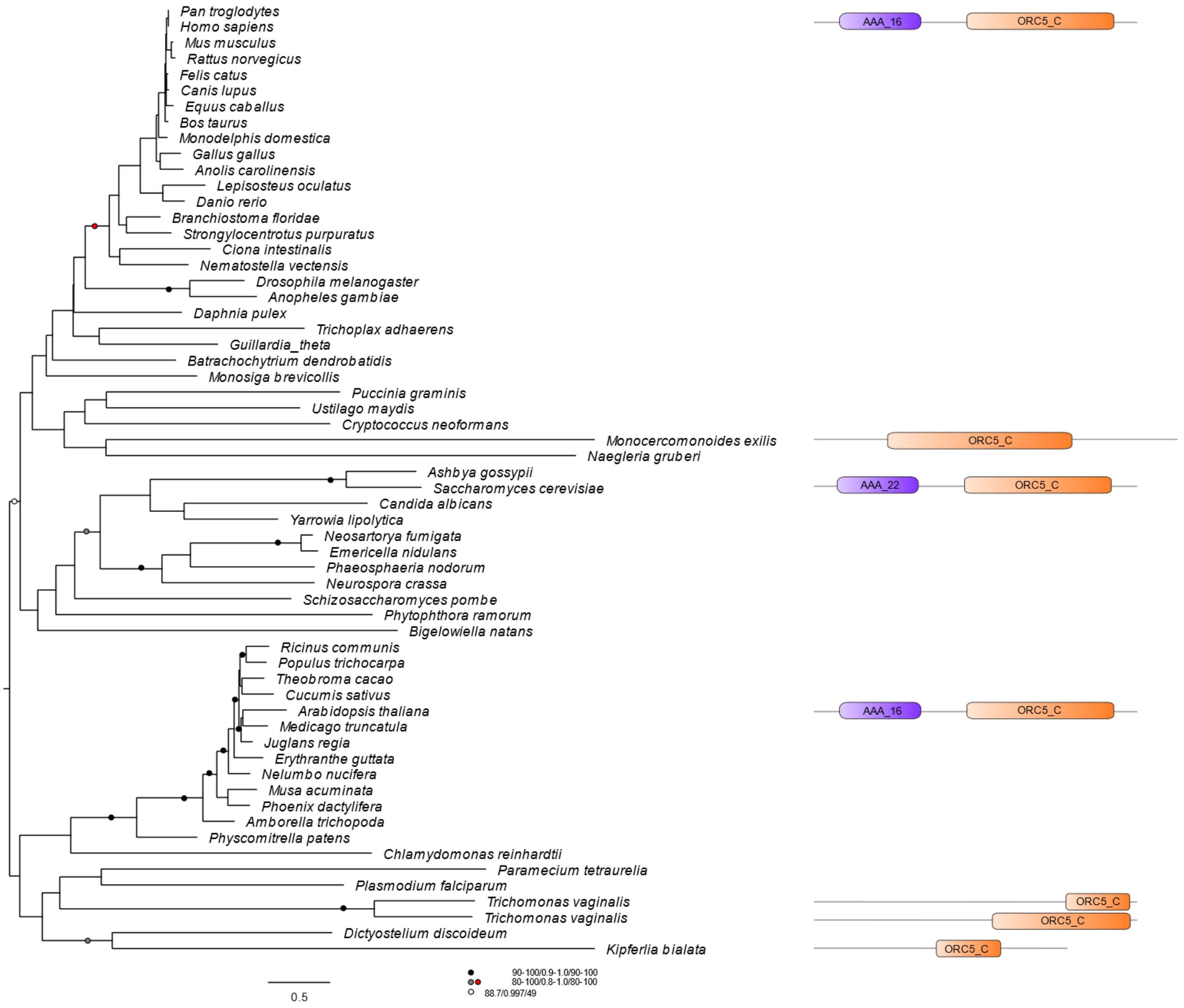

Supplementary Fig. 2 Phylogenetic reconstruction of Orc5 proteins inferred with IQ-TREE[15] under the LG+ C60+F+ Γ model using 1000 ultrafast bootstraps (SH-aLRT support percentage/aBayes/bootstrap). Value ranges for branches are shown by dots, the red dot indicates that the values apply for each node within the clade. The alignment consists of 60 taxa with 422 sites after trimming. For simplicity, only the domain architecture for metamonads, *S. cerevisiae*, *A. thaliana* and *H. sapiens* are depicted on the tree.

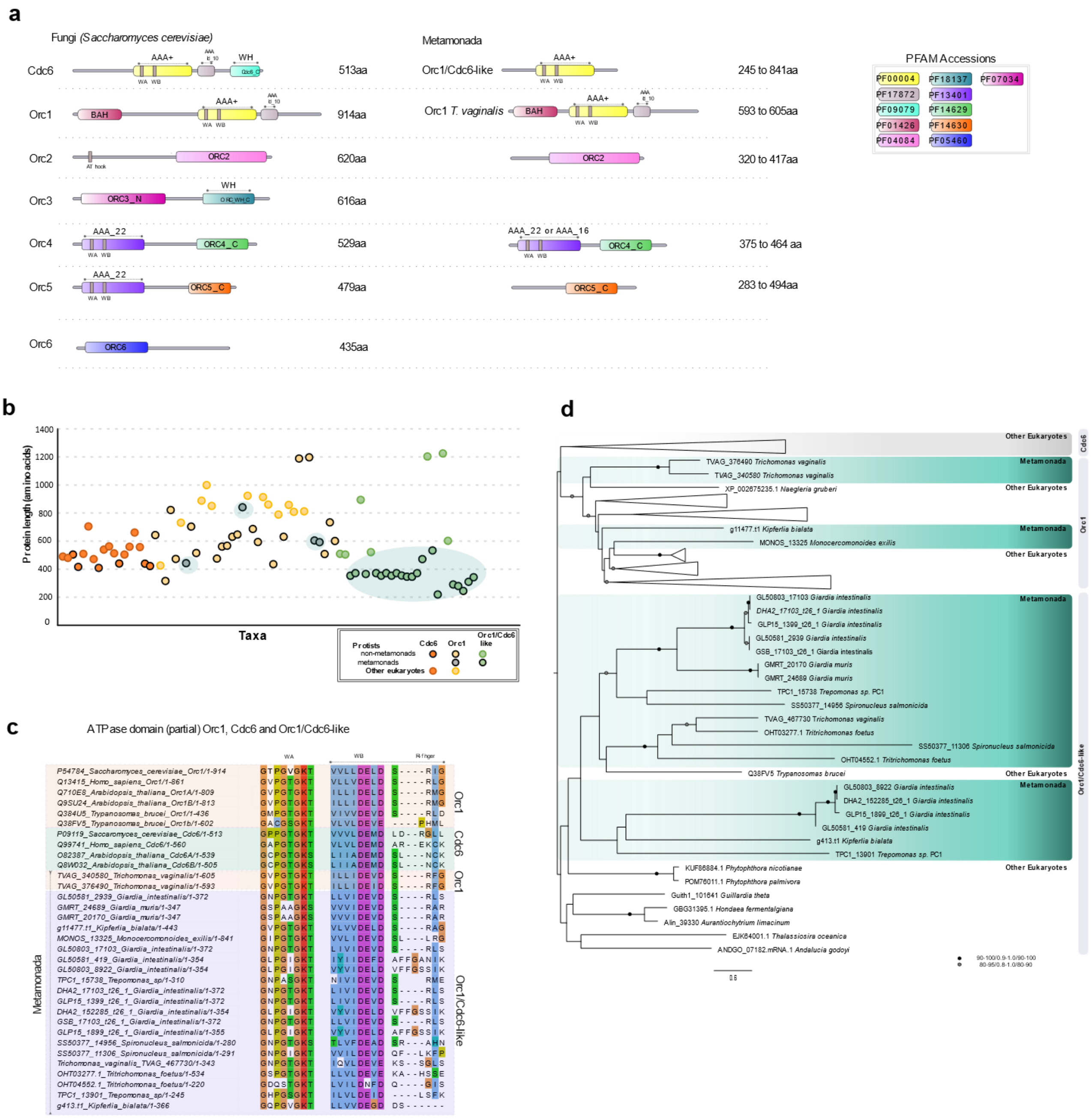

Supplementary Fig. 3 Orc1-6 and Cdc6 proteins. **(a)** Left: typical domain architecture observed for Orc1-6 and Cdc6 in *Saccharomyces cerevisiae*. Right: representative domain architecture of metamonad proteins drawn to reflect the most common protein size. If no species name is given, then the depicted domain structure was found in all of the metamonads where present. Numbers on the right of each depiction correspond to the total protein length or its range in the case of metamonads

(additional information in Supplementary Data 2). **(b)** Comparison of Orc1, Cdc6 and Orc1/Cdc6-like protein lengths across 81 eukaryotes encompassing metamonads and non-metamonads protists (Supplementary Data 6). Metamonad proteins are highlighted with green shaded bubbles in the background. **(c)** Orc1/Cdc6 partial ATPase domain showing Walker A and Walker B motifs including R-finger. Reference species at the top. Multiple sequence alignment was visualized with Jalview[72] using the Clustal colouring scheme. **(d)** Phylogenetic reconstruction of Orc1, Cdc6 and Orc1/Cdc6-like proteins inferred with IQ-TREE[15] under the LG+ C10+F+ Γ model using 1000 ultrafast bootstraps (bootstrap value ranges for branches are shown with black and grey dots). The alignment consists of 81 taxa (Supplementary Data 6) with 367 sites after trimming. Orc1/Cdc6-like proteins do not form a clade with *bona fide* Orc1 and Cdc6 proteins making it impossible to definitively establish whether or not they are orthologs.

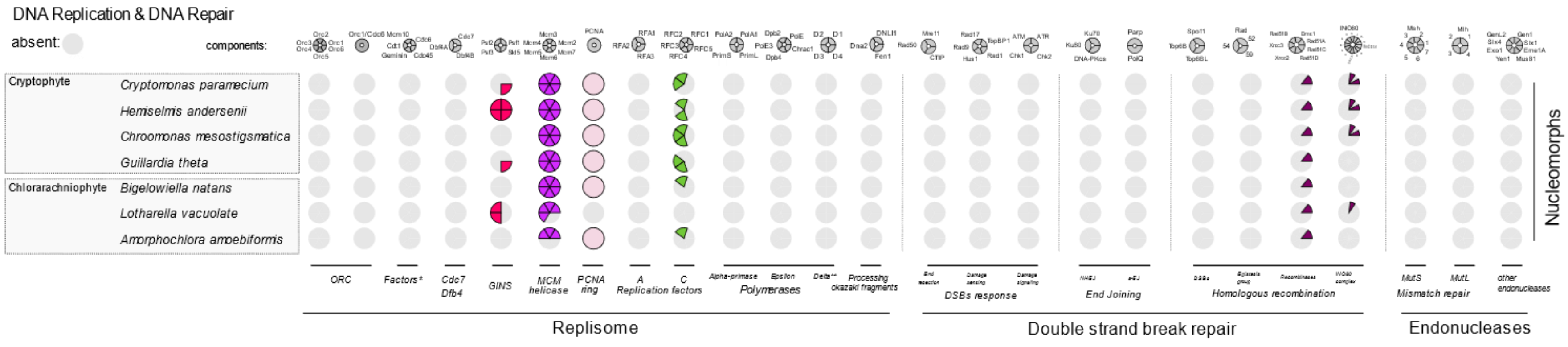

Supplementary Fig. 4 The distribution of core molecular systems of the replisome, double strand break repair and endonucleases in nucleomorph genomes of cryptophyte and chlorarachniophytes.

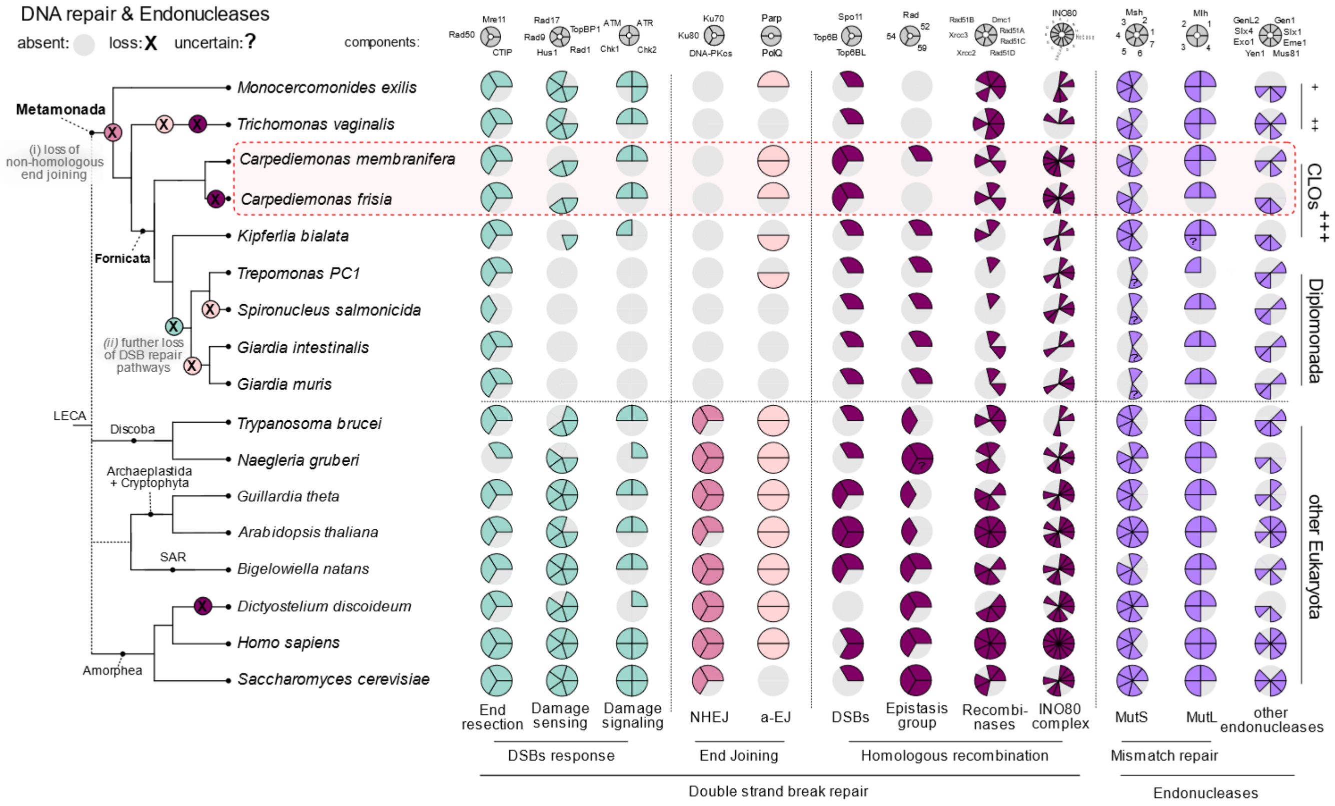

Supplementary Fig. 5 The distribution of core molecular systems of DNA repair across eukaryotic diversity. A schematic global eukaryote phylogeny is shown on the left with classification of the major metamonad lineages indicated. Double strand break repair and endonuclease sets. ***Carpediemonas-Like Organisms. '?' is used in cases where correct orthology was difficult to establish, so the protein name appears with the suffix '-like' in tables.

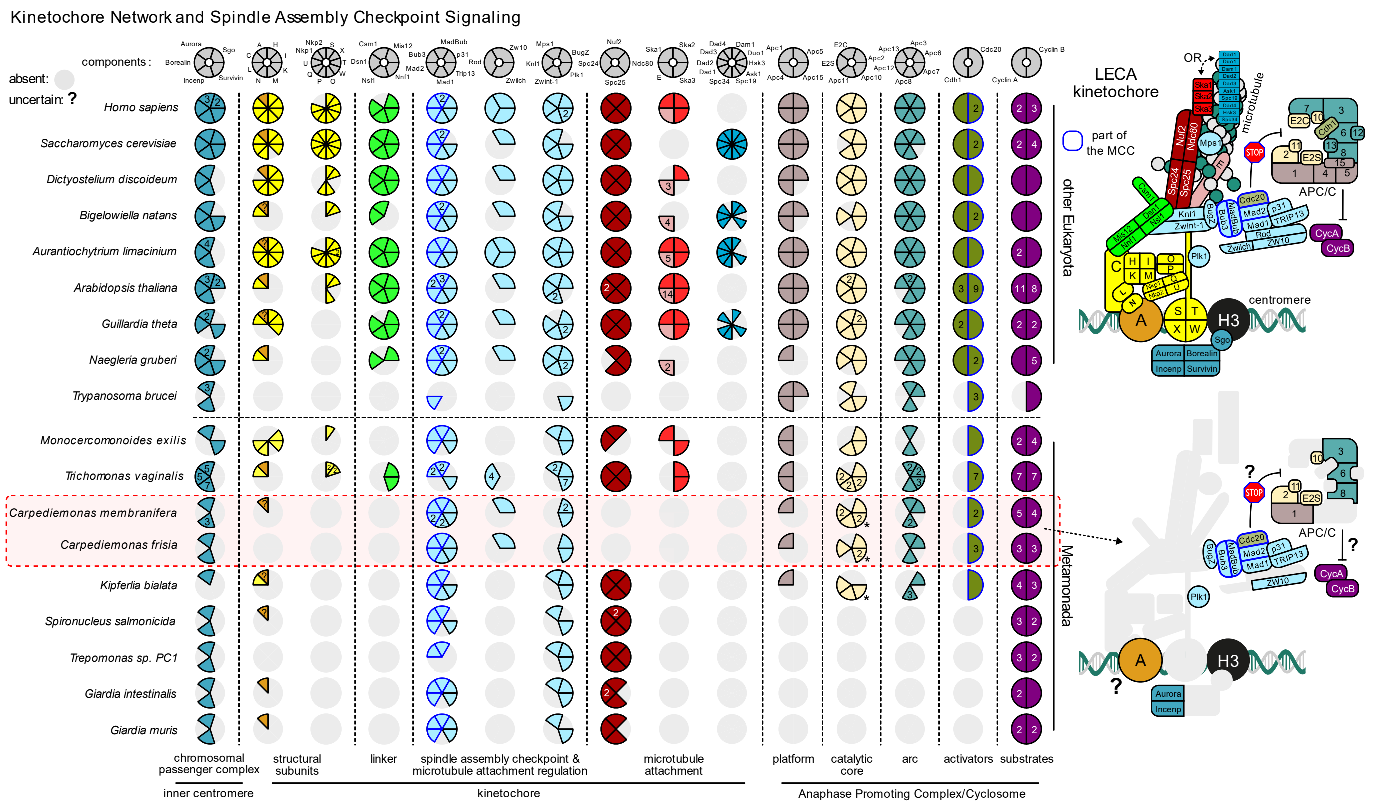

Supplementary Fig. 6 Presence/absence diagram of LECA kinetochore components in eukaryotes, with a greater sampling of metamonads, including *C. membranifera* and *C. frisia*. Left: matrix of presences (coloured) and absences (light grey) of kinetochore, SAC and APC/C proteins that were present in LECA. On top: names of the different subunits; single letters (A-X) indicate Centromere protein A-X (*e.g.*,

CenpA) and numbers, APC/C subunit 1-15 (*e.g.*, Apc1). E2S and E2C, refer to E2 ubiquitin conjugases S and C, respectively. Colour schemes correspond to the kinetochore overview figure on the right and to those used in Figure 3. Right: cartoon of the components of the kinetochore, SAC signalling, the APC/C and its substrates (Cyclin A/B) in LECA and *Carpediemonas* species to indicate the loss of components (light grey shading). Blue lines indicate the presence of proteins that are part of the MCC. Asterisk: Apc10 has three paralogs in *C. membranifera* and two in *C. frisia*. One is the canonical Apc10, the two others are fused to a BTB-Kelch protein of which its closest homologs is a likely adapter for the E3 ubiquitin ligase Cullin 3.

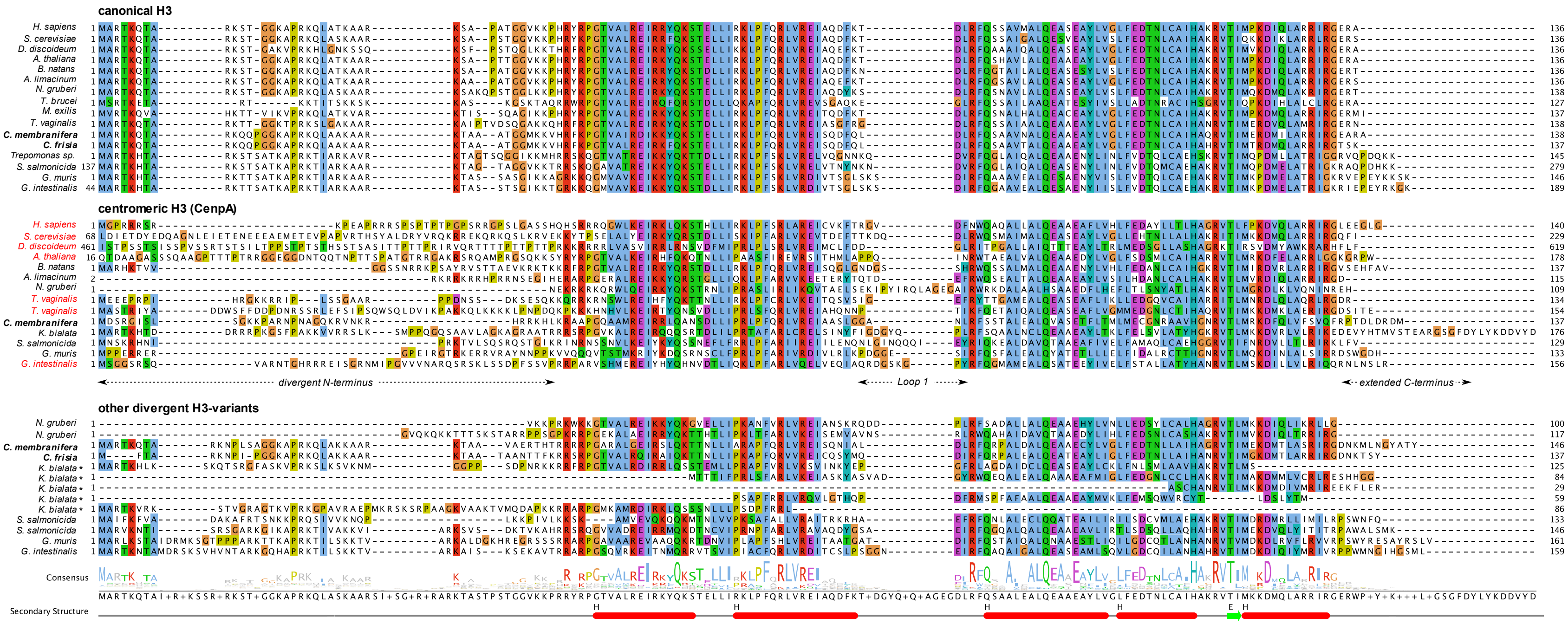

Supplementary Fig. 7 *Carpediemonas* harbours three different types of Histone H3 proteins, a centromere-specific variant (CenpA). Multiple sequence alignment of different Histone H3 variants in eukaryotes and metamonads, including the secondary structure of canonical H3 in humans (pdb: 6ESF_A). CenpA orthologs are characterized by extended amino and carboxy termini and a large L1 loop. Red names in the CenpA panel indicate for which species centromere/kinetochore localization has been confirmed. In addition to CenpA and canonical Histone H3-variants, multiple eukaryotes, including *C. membranifera* and *C. frisia*, harbour other divergent H3 variants. Such divergent variants make the annotation of Histone H3 homologs ambiguous (see Asterisks; incomplete sequences). Multiple sequence alignments were visualized with Jalview[72], using the Clustal colour scheme. Asterisks indicate two potential CenpA candidates in *T. vaginalis*

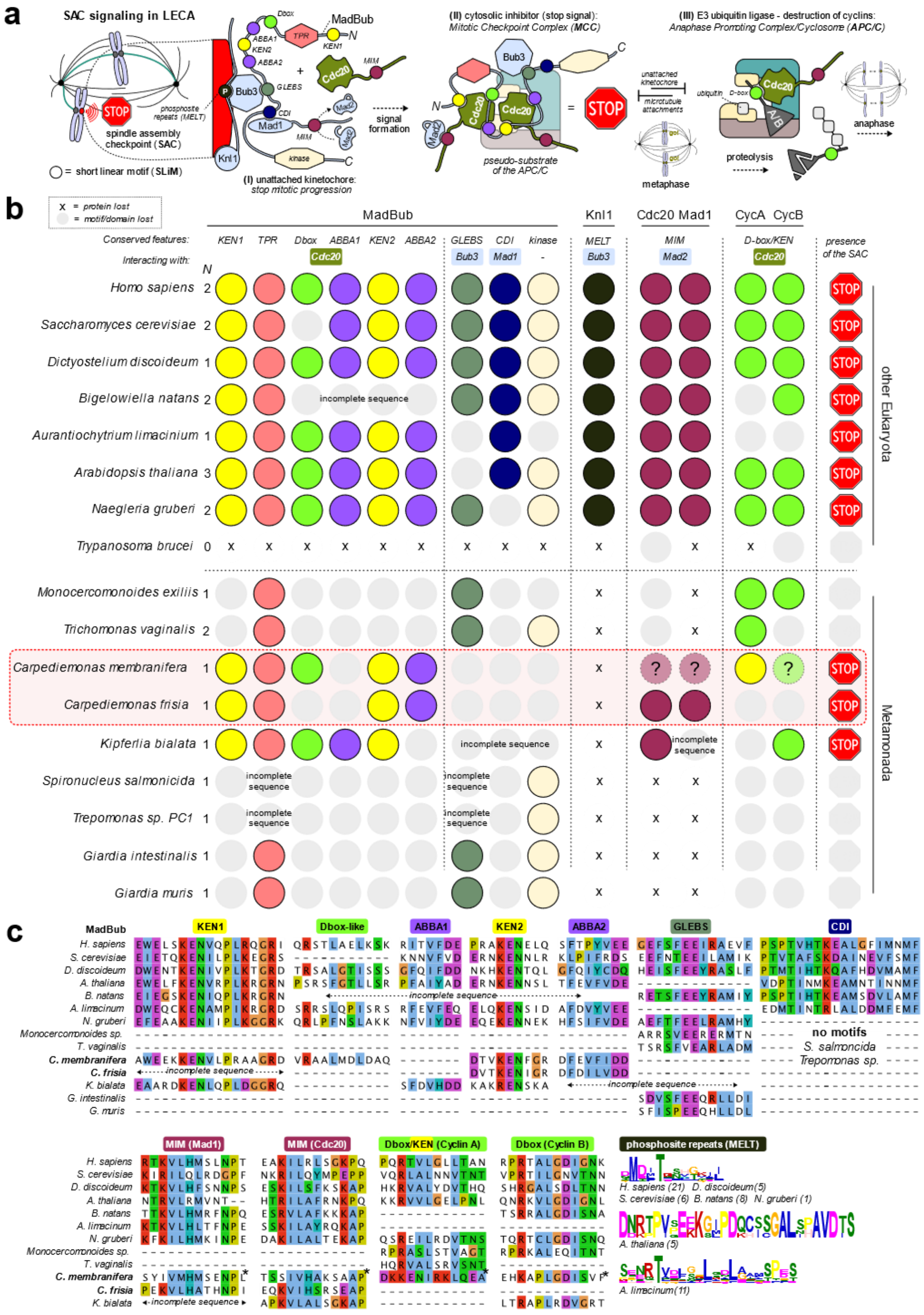

Supplementary Fig. 8 Likely presence of SAC signalling in *Carpediemonas*. **(a)** Short linear motifs form the basis of SAC signalling. During prometaphase, unattached kinetochores catalyse the production of inhibitor of the cell cycle machinery, a phenomenon known as the SAC[73]. (I) The main

protein scaffold of SAC signalling is the kinase MadBub (paralogs Mad3/Bub1 exist in eukaryotes), which consist of many short linear motifs (SLiMs) that mediate the interaction of SAC components and the APC/C (light blue)[74,75]. MadBub itself is recruited to the kinetochore through interaction with Bub3 (GLEBS), which on its turn binds repeated phosphomotifs in Knl1[76-78]. The CDI or CMI motif aids to recruit Mad1[79-81], which has a Mad2-interaction Motif (MIM) that mediated the kinetochore-dependent conversion of open-Mad2 to Mad2 in a closed conformation[82]. (II) Mad2, MadBub, Bub3 and 2x Cdc20 (APC/C co-activator) form the mitotic checkpoint complex (MCC) and block the APC/C[75,83,84]. MadBub contains 3 different APC/C degrons (D-box, KEN-box and ABBA motif)[74] that direct its interaction with 2x Cdc20s and effectively make the MCC a pseudo substrate of the APC/C. (III) Increasing amounts of kinetochore-microtubule attachments silence the production of the MCC at kinetochores and the APC/C is released. Cdc20 now presents its substrates Cyclin A and Cyclin B (some eukaryotes have other substrates as well, but they are not universally conserved) for ubiquitination and subsequent degradation through recognition of a Dbox motif[85]. Chromosome segregation will now be initiated (anaphase). **(b)** Presence/absence matrix of motifs involved in SAC signalling in a selection of Eukaryotes and Metamonads, including *C. membranifera* and *C. frisia*. Colours correspond to the motifs in panel a, light grey indicates motif loss. *N* signifies the number of MadBub homologs that are present in each species. ‘Incomplete’ points to sequences that were found to be incomplete due to gaps in the genome assembly. Question marks indicate the uncertainty in the presence of that particular motif. Although Metamonads have all four MCC components (Mad2, Bub3, MadBub and Cdc20), most homologs do not contain the motifs to elicit a canonical SAC signalling and it is therefore likely that they do not have a SAC response. Exceptions are *C membranifera*, *C. frisia* and *Kipferlia bialata*. They retained the N-terminal KEN-boxes and one ABBA motif, which are involved in the binding of two Cdc20s and a Mad2-interaction motif (MIM) in Mad1 and Cdc20. **(c)** Multiple sequence alignments of the motifs from panel A and B. Coloured

motif boxes correspond to panel a and b. Multiple sequence alignments were visualized with Jalview[72], using the Clustal colouring scheme. Asterisks indicate ambiguous motifs in *Carpediemonas membranifera*.

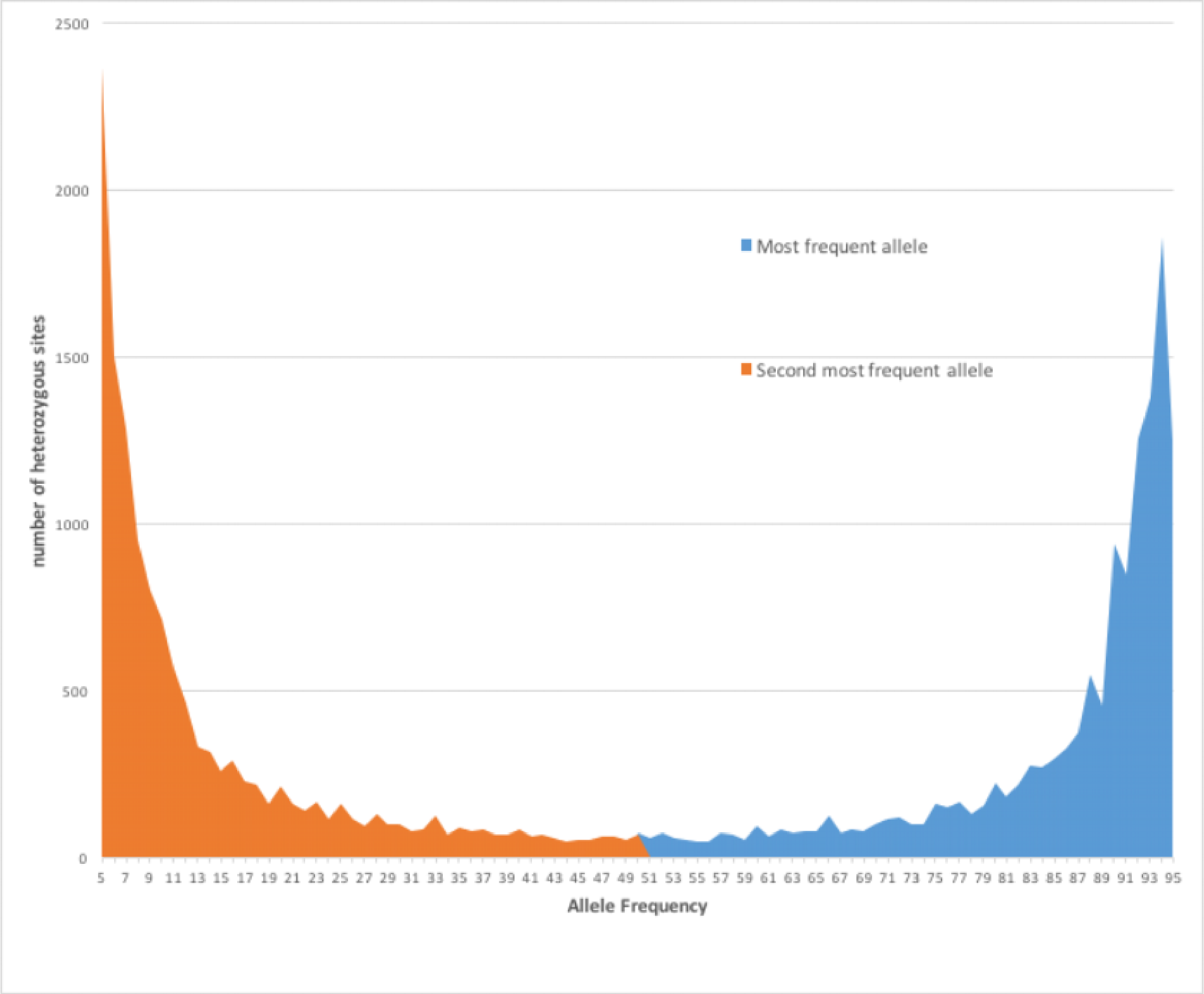

Supplementary Fig. 9 Histogram showing the frequency distribution of single nucleotide variants in the genome of *C. membranifera*. Diagram showing the typical distribution of a haploid genome.

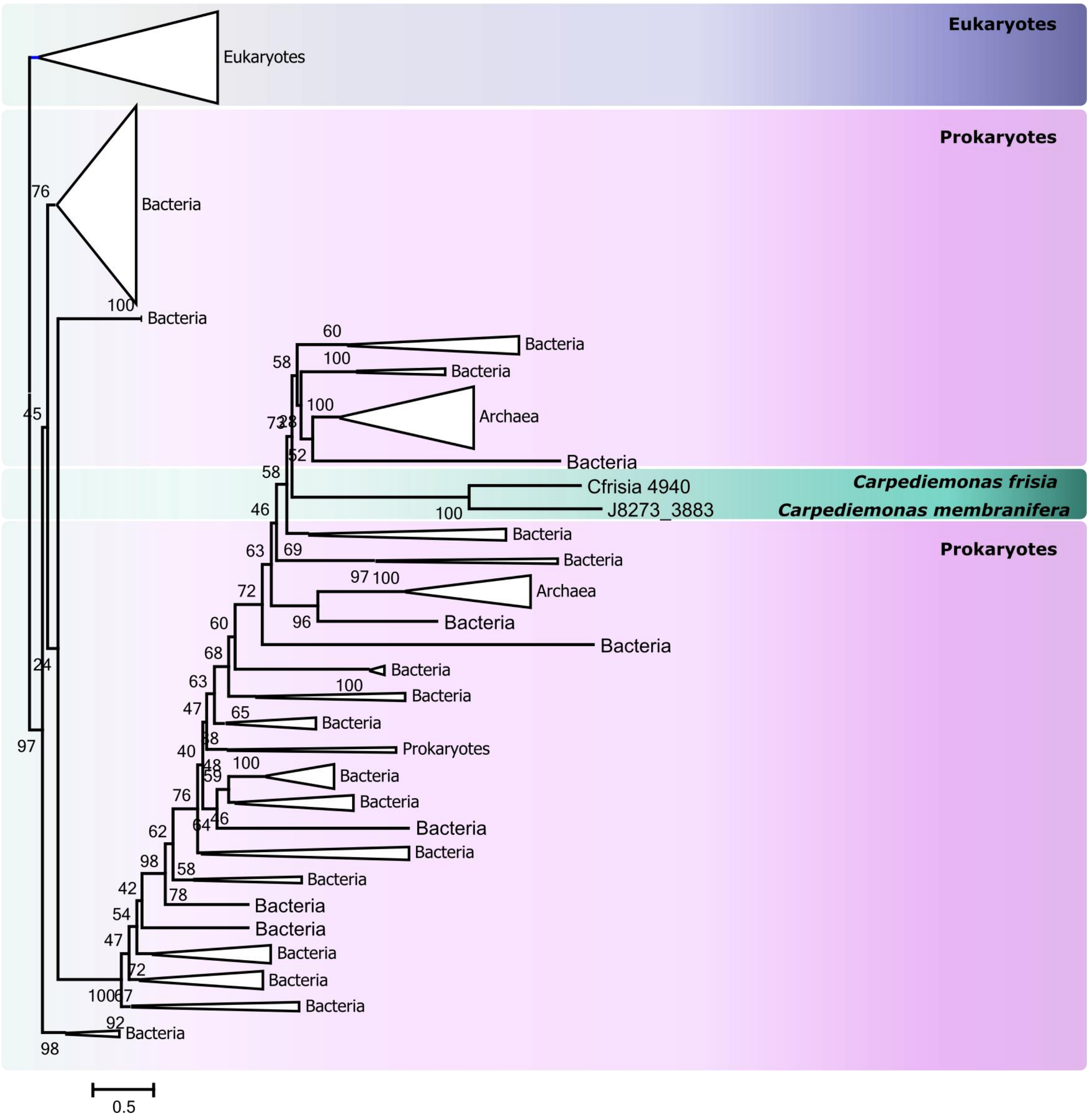

Supplementary Fig. 10 Maximum likelihood reconstruction of Endonuclease IV. The unrooted tree contains eukaryotic and prokaryotic Endo IV sequences, showing *Carpediemonas* sequences emerging within bacterial proteins. The tree was inferred with IQ-TREE under the LG+I+C20 model with 1000 ultrafast bootstraps; alignment length was 276. Scale bar shows the inferred number of amino acid substitutions per site.

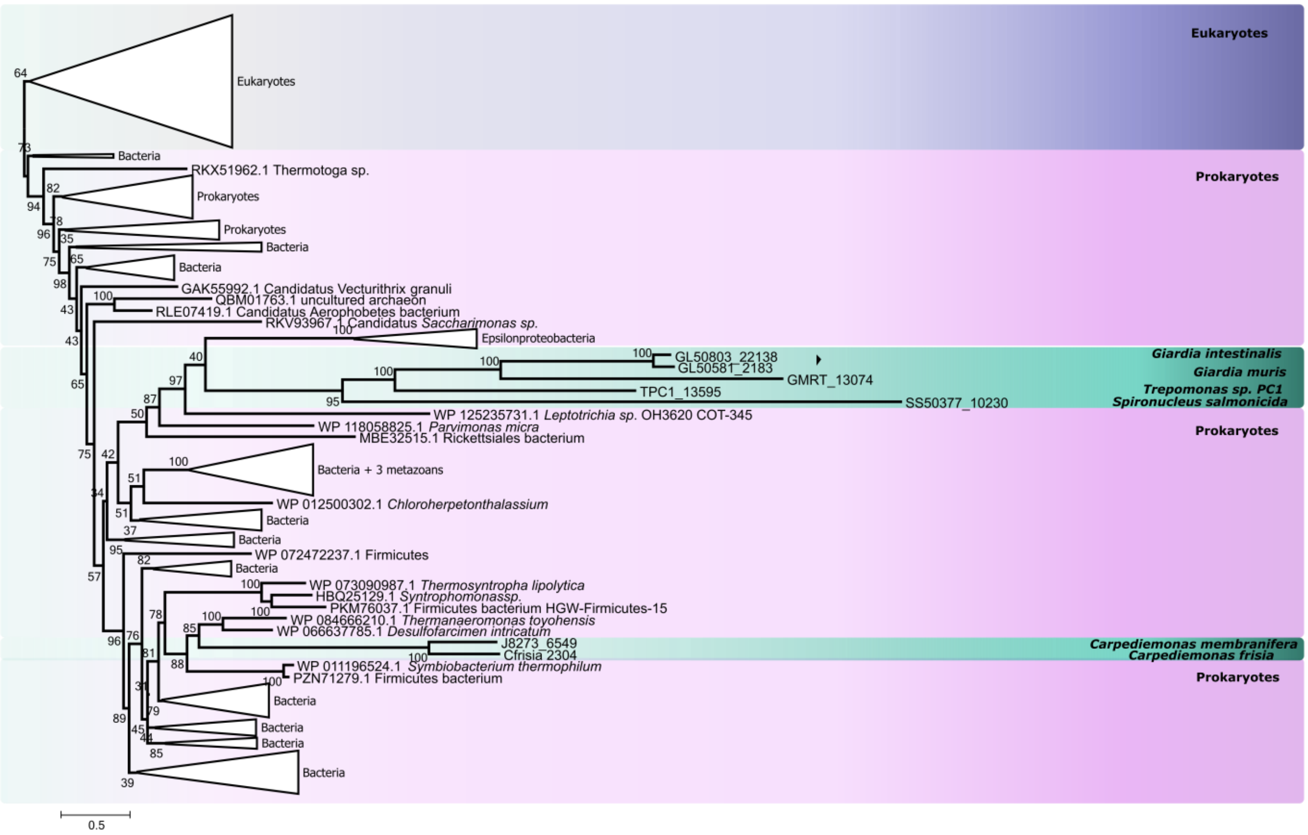

Supplementary Fig. 11 Maximum likelihood reconstruction of RarA. The unrooted tree contains eukaryotic and prokaryotic sequences, showing *Carpediemonas* sequences emerging within bacterial proteins. The tree was inferred with IQ-TREE under the LG+I+C20 model with 1000 ultrafast bootstraps; alignment length was 414. Scale bar shows the inferred number of amino acid substitutions per site.

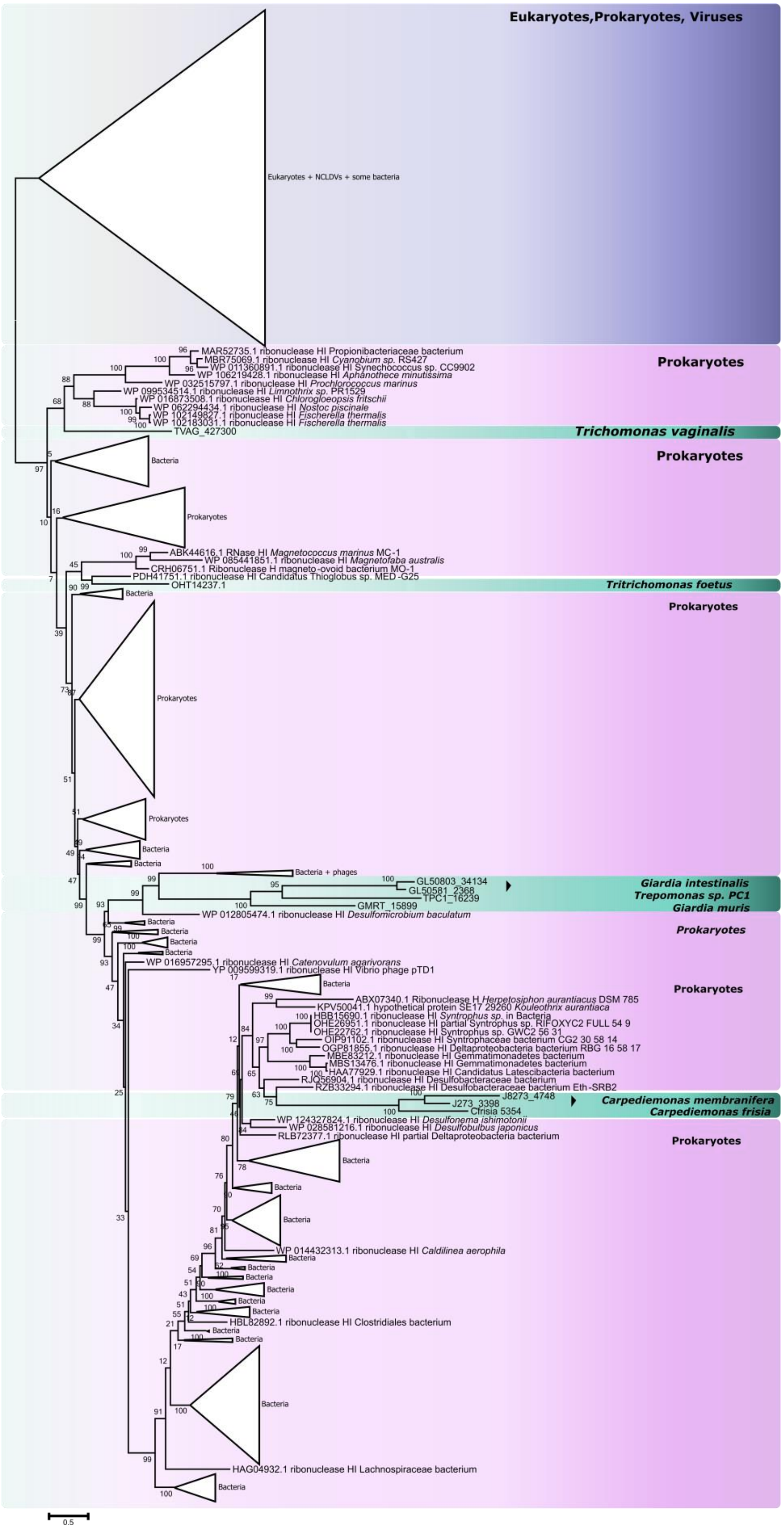
Eukaryotes,Prokaryotes, Viruses
Eukaryotes + NCLDVs + some bacteria
Prokaryotes
MAR52735.1 ribonuclease HI Propionibacteriaceae bacterium
MBR75069.1 ribonuclease HI Cyanobium sp. RS427
WP 011360891.1 ribonuclease HI Synechococcus sp. CC9902
WP 106219428.1 ribonuclease HI Aphanothece minutissima
WP 032515797.1 ribonuclease HI Prochlorococcus marinus
WP 099534514.1 ribonuclease HI Limnothrix sp. PR1529
WP 016873508.1 ribonuclease HI Chlorogloeopsis fritschii
WP 062294434.1 ribonuclease HI Nostoc piscinale
WP 102149827.1 ribonuclease HI Fischerella thermalis
WP 102183031.1 ribonuclease HI Fischerella thermalis
TVAG_427300
Trichomonas vaginalis
Prokaryotes
Bacteria
Prokaryotes
ABK44616.1 RNase HI Magnetococcus marinus MC-1
WP 085441851.1 ribonuclease HI Magnetofaba australis
CRH06751.1 Ribonuclease H magneto-ovoid bacterium MO-1
PDH41751.1 ribonuclease HI Candidatus Thioglobus sp. MED-G25
OHT14237.1
Tritrichomonas foetus
Prokaryotes
Bacteria
Prokaryotes
Prokaryotes
Bacteria
Bacteria
Bacteria + phages
GL50803_34134
GL50581_2368
TPC1_16239
GMRT_15899
Giardia intestinalis
Trepomonas sp. PC1
Giardia muris
WP 012805474.1 ribonuclease HI Desulfomicrobium baculatum
Prokaryotes
Bacteria
Bacteria
Bacteria
Bacteria
WP 016957295.1 ribonuclease HI Catenovulum agarivorans
YP 009599319.1 ribonuclease HI Vibrio phage pTD1
Prokaryotes
Bacteria
ABX07340.1 Ribonuclease H Herpetosiphon aurantiacus DSM 785
KPV50041.1 hypothetical protein SE17 29260 Kouleothrix aurantiaca
HBB15690.1 ribonuclease HI Syntrophus sp. in Bacteria
OHE26951.1 ribonuclease HI partial Syntrophus sp. RIFOXYC2 FULL 54 9
OHE22762.1 ribonuclease HI Syntrophus sp. GWC2 56 31
OIP91102.1 ribonuclease HI Syntrophaceae bacterium CG2 30 58 14
OGP81855.1 ribonuclease HI Deltaproteobacteria bacterium RBG 16 58 17
MBE83212.1 ribonuclease HI Gemmatimonadetes bacterium
MBS13476.1 ribonuclease HI Gemmatimonadetes bacterium
HAA77929.1 ribonuclease HI Candidatus Latescibacteria bacterium
RJQ56904.1 ribonuclease HI Desulfobacteraceae bacterium
RZB33294.1 ribonuclease HI Desulfobacteraceae bacterium Eth-SRB2
J8273_4748
J273_3398
Cfrisia 5354
Carpediemonas membranifera
Carpediemonas frisia
WP 124327824.1 ribonuclease HI Desulfonema ishimotonii
WP 028581216.1 ribonuclease HI Desulfobulbus japonicus
RLB72377.1 ribonuclease HI partial Deltaproteobacteria bacterium
Prokaryotes
Bacteria
Bacteria
Bacteria
WP 014432313.1 ribonuclease HI Caldilinea aerophila
Bacteria
Bacteria
Bacteria
Bacteria
Bacteria
Bacteria
HBL82892.1 ribonuclease HI Clostridiales bacterium
Bacteria
Bacteria
Bacteria
HAG04932.1 ribonuclease HI Lachnospiraceae bacterium
Bacteria
0.5

Supplementary Fig. 12 Maximum likelihood reconstruction of RNAse H1. *Carpediemonas* RarA-like proteins emerge within bacterial proteins. Parabasalia and Diplomonada proteins highlighting the proteins have been acquired in different events. The tree was inferred with IQ-TREE under the LG+I+G+C20 model with 1000 ultrafast bootstraps; alignment length was 149. Scale bar shows the inferred number of amino acid substitutions per site.

E. Supplementary references

1 Wick, R. R., Judd, L. M., Gorrie, C. L. & Holt, K. E. Completing bacterial genome assemblies with multiplex MinION sequencing. *Microb. Genom.* **3**, e000132-e000132 (2017).

2 Lin, Y. et al. Assembly of long error-prone reads using de Bruijn graphs. *Proc. Natl. Acad. Sci. U.S.A* **113**, E8396-E8405 (2016).

3 Loman, N. J., Quick, J. & Simpson, J. T. A complete bacterial genome assembled de novo using only nanopore sequencing data. *Nat. Methods* **12**, 733-735 (2015).

4 Wick, R. R., Judd, L. M., Gorrie, C. L. & Holt, K. E. Unicycler: resolving bacterial genome assemblies from short and long sequencing reads. *PLoS Comput. Biol.* **13**, e1005595 (2017).

5 Walker, B. J. et al. Pilon: an integrated tool for comprehensive microbial variant detection and genome assembly improvement. *PLoS One* **9**, e112963 (2014).

6 Langmead, B. & Salzberg, S. L. Fast gapped-read alignment with Bowtie 2. *Nat. Methods* **9**, 357-359 (2012).

7 Camacho, C. et al. BLAST+: architecture and applications. *BMC Bioinformatics* **10**, 421 (2009).

8 Li, H. et al. The sequence alignment/map format and SAMtools. *Bioinformatics* **25**, 2078-2079 (2009).

9 Pedersen, B. S. & Quinlan, A. R. Mosdepth: quick coverage calculation for genomes and exomes. *Bioinformatics* **34**, 867-868 (2018).

10 Waterhouse, R. M. et al. BUSCO applications from quality assessments to gene prediction and phylogenomics. *Mol. Biol. Evol.* **35**, 543-548 (2018).

11 Leger, M. M. et al. Organelles that illuminate the origins of *Trichomonas* hydrogenosomes and *Giardia* mitosomes. *Nat Ecol Evol* **1**, 0092 (2017).

12 Mi, H. et al. PANTHER version 11: expanded annotation data from Gene Ontology and Reactome pathways, and data analysis tool enhancements. *Nucleic Acids Res.* **45**, D183-D189 (2017).

13 Eddy, S. R. Accelerated profile HMM searches. *PLoS Comp. Biol.* **7**, e1002195 (2011).

14 Yamada, K. D., Tomii, K. & Katoh, K. Application of the MAFFT sequence alignment program to large data-reexamination of the usefulness of chained guide trees. *Bioinformatics* **32**, 3246-3251 (2016).

15 Nguyen, L. T., Schmidt, H. A., von Haeseler, A. & Minh, B. Q. IQ-TREE: a fast and effective stochastic algorithm for estimating maximum-likelihood phylogenies. *Mol. Biol. Evol.* **32**, 268-274 (2015).

16 Huerta-Cepas, J., Serra, F. & Bork, P. ETE 3: reconstruction, analysis, and visualization of phylogenomic data. *Mol. Biol. Evol.* **33**, 1635-1638 (2016).

17 Carlton, J. M. et al. Draft genome sequence of the sexually transmitted pathogen *Trichomonas vaginalis*. *Science* **315**, 207-212 (2007).

18 Karnkowska, A. et al. A eukaryote without a mitochondrial organelle. *Curr. Biol.* **26**, 1274-1284 (2016).

19 Hamann, E. et al. Syntrophic linkage between predatory *Carpediemonas* and specific prokaryotic populations. *ISME J* **11**, 1205-1217 (2017).

20 Tanifuji, G. et al. The draft genome of *Kipferlia bialata* reveals reductive genome evolution in fornicate parasites. *PLoS One* **13**, e0194487 (2018).

21 Morrison, H. G. et al. Genomic minimalism in the early diverging intestinal parasite *Giardia lamblia*. *Science* **317** (2007).

22 Franzen, O. et al. Draft genome sequencing of *Giardia intestinalis* assemblage B isolate GS: is human giardiasis caused by two different species? *PLoS Pathog* **5**, e1000560 (2009).

23 Xu, F. et al. The compact genome of *Giardia muris* reveals important steps in the evolution of intestinal protozoan parasites. *Microb. Genom.* (2020).

24 Xu, F., Jex, A. & Svard, S. G. A chromosome-scale reference genome for *Giardia intestinalis* WB. *Sci Data* **7**, 38 (2020).

25 Xu, F. et al. The genome of *Spironucleus salmonicida* highlights a fish pathogen adapted to fluctuating environments. *PLoS Genet.* **10**, e1004053 (2014).

26 Xu, F. et al. On the reversibility of parasitism: adaptation to a free-living lifestyle via gene acquisitions in the diplomonad *Trepomonas sp.* PC1. *BMC Biol.* **14**, 62 (2016).

27 International Human Genome Sequencing, C. Finishing the euchromatic sequence of the human genome. *Nature* **431**, 931-945 (2004).

28 The Arabidopsis Genome, I. Analysis of the genome sequence of the flowering plant *Arabidopsis thaliana*. *Nature* **408**, 796-815 (2000).

29 Eichinger, L. et al. The genome of the social amoeba *Dictyostelium discoideum*. *Nature* **435**, 43-57 (2005).

30 Berriman, M. et al. The genome of the African trypanosome *Trypanosoma brucei*. *Science* **309**, 416-422 (2005).

31 Fritz-Laylin, L. K. et al. The genome of *Naegleria gruberi* illuminates early eukaryotic versatility. *Cell* **140**, 631-642 (2010).

32 Curtis, B. A. et al. Algal genomes reveal evolutionary mosaicism and the fate of nucleomorphs. *Nature* **492**, 59-65 (2012).

33 Corradi, N., Pombert, J. F., Farinelli, L., Didier, E. S. & Keeling, P. J. The complete sequence of the smallest known nuclear genome from the microsporidian *Encephalitozoon intestinalis*. *Nat Commun* **1**, 77 (2010).

34 Katinka, M. D. et al. Genome sequence and gene compaction of the eukaryote parasite *Encephalitozoon cuniculi*. *Nature* **414**, 450-453 (2001).

35 Heinz, E. et al. The genome of the obligate intracellular parasite *Trachipleistophora hominis*: new insights into microsporidian genome dynamics and reductive evolution. *PLoS Pathog* **8**, e1002979 (2012).

36 Steenwyk, J. L. et al. Extensive loss of cell-cycle and DNA repair genes in an ancient lineage of bipolar budding yeasts. *PLoS Biol.* **17**, e3000255 (2019).

37 Sternes, P. R., Lee, D., Kutyna, D. R. & Borneman, A. R. Genome sequences of three species of *Hanseniaspora* isolated from spontaneous wine fermentations. *Genome Announc* **4**, e01287-01216 (2016).

38 Riley, R. et al. Comparative genomics of biotechnologically important yeasts. *Proc. Natl. Acad. Sci. U.S.A.* **113**, 9882-9887 (2016).

39 Benchimol, M. et al. Draft genome sequence of *Tritrichomonas foetus* Strain K. *Genome Announc.* **5**, e00195-00117 (2017).

40 Lane, C. E. et al. Nucleomorph genome of *Hemiselmis andersenii* reveals complete intron loss and compaction as a driver of protein structure and function. *Proc. Natl. Acad. Sci. U.S.A.* **104**, 19908-19913 (2007).

41 Tanifuji, G. et al. Complete nucleomorph genome sequence of the nonphotosynthetic alga *Cryptomonas paramecium* reveals a core nucleomorph gene set. *Genome Biol. Evol.* **3**, 44-54 (2011).

42 Moore, C. E., Curtis, B., Mills, T., Tanifuji, G. & Archibald, J. M. Nucleomorph genome sequence of the cryptophyte alga *Chroomonas mesostigmatica* CCMP1168 reveals lineage-specific gene loss and genome complexity. *Genome Biol. Evol.* **4**, 1162-1175 (2012).

43 Douglas, S. et al. The highly reduced genome of an enslaved algal nucleus. *Nature* **410**, 1091-1096 (2001).

44 Suzuki, S., Shirato, S., Hirakawa, Y. & Ishida, K. Nucleomorph genome sequences of two chlorarachniophytes, *Amorphochlora amoebiformis* and *Lotharella vacuolata*. *Genome Biol. Evol.* **7**, 1533-1545 (2015).

45 Gilson, P. R. et al. Complete nucleotide sequence of the chlorarachniophyte nucleomorph: nature's smallest nucleus. *Proc. Natl. Acad. Sci. U.S.A* **103**, 9566-9571 (2006).

46 Ying, H. et al. Comparative genomics reveals the distinct evolutionary trajectories of the robust and complex coral lineages. *Genome Biol.* **19**, 175-175 (2018).

47 Voolstra, C. et al. The ReFuGe 2020 Consortium—using “omics” approaches to explore the adaptability and resilience of coral holobionts to environmental change. *Front. Mar. Sci.* **2** (2015).

48 Brown, M. W. et al. Phylogenomics places orphan protistan lineages in a novel eukaryotic super-group. *Genome Biol. Evol.* **10**, 427-433 (2018).

49 Keeling, P. J. et al. The Marine Microbial Eukaryote Transcriptome Sequencing Project (MMETSP): illuminating the functional diversity of eukaryotic life in the oceans through transcriptome sequencing. *PLoS Biol.* **12**, e1001889 (2014).

50 Criscuolo, A. & Gribaldo, S. BMGE (Block Mapping and Gathering with Entropy): a new software for selection of phylogenetic informative regions from multiple sequence alignments. *BMC Evol. Biol.* **10**, 210 (2010).

51 Tromer, E. C., van Hooff, J., Kops, G. & Snel, B. Mosaic origin of the eukaryotic kinetochore. *Proc. Natl. Acad. Sci.*, 201821945 (2019).

52 Pedroza-Garcia, J. A. et al. Function of the plant DNA polymerase epsilon in replicative stress sensing, a genetic analysis. *Plant Physiol.* **173**, 1735-1749 (2017).

53 Johansson, E. & Dixon, N. Replicative DNA polymerases. *Cold Spring Harb. Perspect. Biol.* **5** (2013).

54 Jedrychowska, M. et al. Defects in the GINS complex increase the instability of repetitive sequences via a recombination-dependent mechanism. *PLoS Genet.* **15**, e1008494 (2019).

55 Cheng, E. et al. Genome rearrangements caused by depletion of essential DNA replication proteins in *Saccharomyces cerevisiae*. *Genetics* **192**, 147-160 (2012).

56 Le May, N., Egly, J. M. & Coin, F. True lies: the double life of the nucleotide excision repair factors in transcription and DNA repair. *J Nucleic Acids* **2010**, 616342 (2010).

57 Einarsson, E., Svard, S. G. & Troell, K. UV irradiation responses in *Giardia intestinalis*. *Exp. Parasitol.* **154**, 25-32 (2015).

58 Snowden, T., Acharya, S., Butz, C., Berardini, M. & Fishel, R. hMSH4-hMSH5 recognizes Holliday Junctions and forms a meiosis-specific sliding clamp that embraces homologous chromosomes. *Mol. Cell* **15**, 437-451 (2004).

59 Lee, S. D., Surtees, J. A. & Alani, E. *Saccharomyces cerevisiae* MSH2-MSH3 and MSH2-MSH6 complexes display distinct requirements for DNA binding domain I in mismatch recognition. *J. Mol. Biol.* **366**, 53-66 (2007).

60 Karnkowska, A. et al. The Oxymonad genome displays canonical eukaryotic complexity in the absence of a mitochondrion. *Mol. Biol. Evol.* **36**, 2292-2312 (2019).

61 Dang, H. Q. & Li, Z. The Cdc45·Mcm2-7·GINS protein complex in trypanosomes regulates DNA replication and interacts with two Orc1-like proteins in the origin recognition complex. *J. Biol. Chem.* **286**, 32424-32435 (2011).

62 Onuma, R., Mishra, N. & Miyagishima, S. Y. Regulation of chloroplast and nucleomorph replication by the cell cycle in the cryptophyte *Guillardia theta*. *Sci. Rep.* **7**, 2345 (2017).

63 Suzuki, S., Ishida, K. & Hirakawa, Y. Diurnal transcriptional regulation of endosymbiotically derived genes in the chlorarachniophyte *Bigelowiella natans*. *Genome Biol. Evol.* **8**, 2672-2682 (2016).

64 Romero, H. et al. Single molecule tracking reveals functions for RarA at replication forks but also independently from replication during DNA repair in *Bacillus subtilis*. *Sci. Rep.* **9**, 1997 (2019).

65 Yoshimura, A., Seki, M. & Enomoto, T. The role of WRNIP1 in genome maintenance. *Cell Cycle* **16**, 515-521 (2017).

66 Cerritelli, S. et al. Failure to produce mitochondrial DNA results in embryonic lethality in RNAseH1 null mice. *Mol. Cell* **11**, 807-815 (2003).

67 Nowotny, M. et al. Specific recognition of RNA/DNA hybrid and enhancement of human RNase H1 activity by HBD. *EMBO J.* **27**, 1172-1181 (2008).

68 Cerritelli, S. M. & Crouch, R. J. Ribonuclease H: the enzymes in eukaryotes. *FEBS J.* **276**, 1494-1505 (2009).

69 Saary, P., Mitchell, A. L. & Finn, R. D. Estimating the quality of eukaryotic genomes recovered from metagenomic analysis with EukCC. *Genome Biol.* **21**, 244 (2020).

70 Hanschen, E., Hovde, B. & Starkenburg, S. An evaluation of methodology to determine algal genome completeness. *Algal Res.* **51**, 102019 (2020).

71 Rhie, A., Walenz, B. P., Koren, S. & Phillippy, A. M. Merqury: reference-free quality, completeness, and phasing assessment for genome assemblies. *Genome Biol.* **21**, 245 (2020).

72 Waterhouse, A. M., Procter, J. B., Martin, D. M., Clamp, M. & Barton, G. J. Jalview Version 2--a multiple sequence alignment editor and analysis workbench. *Bioinformatics* **25**, 1189-1191 (2009).

73 Musacchio, A. The molecular biology of spindle assembly checkpoint signaling dynamics. *Curr. Biol.* **25**, R1002-R1018 (2015).

74 Alfieri, C., Zhang, S. & Barford, D. Visualizing the complex functions and mechanisms of the anaphase promoting complex/cyclosome (APC/C). *Open Biol.* **7** (2017).

75 Tromer, E. C., Bade, D., Snel, B. & Kops, G. J. Phylogenomics-guided discovery of a novel conserved cassette of short linear motifs in BubR1 essential for the spindle checkpoint. *Open Biol.* **6** (2016).

76 Vleugel, M. et al. Arrayed BUB recruitment modules in the kinetochore scaffold KNL1 promote accurate chromosome segregation. *J. Cell Biol.* **203**, 943-955 (2013).

77 Shepperd, L. A. et al. Phosphodependent recruitment of Bub1 and Bub3 to Spc7/KNL1 by Mph1 kinase maintains the spindle checkpoint. *Curr. Biol.* **22**, 891-899 (2012).

78 Tromer, E. C., Snel, B. & Kops, G. Widespread recurrent patterns of rapid repeat evolution in the kinetochore scaffold KNL1. *Genome Biol. Evol.* **7**, 2383-2393 (2015).

79 Moyle, M. W. et al. A Bub1-Mad1 interaction targets the Mad1-Mad2 complex to unattached kinetochores to initiate the spindle checkpoint. *J. Cell Biol.* **204**, 647-657 (2014).

80 Ji, Z., Gao, H., Jia, L., Li, B. & Yu, H. A sequential multi-target Mps1 phosphorylation cascade promotes spindle checkpoint signaling. *Elife* **6** (2017).

81 Zhang, G. et al. Bub1 positions Mad1 close to KNL1 MELT repeats to promote checkpoint signalling. *Nat. Commun.* **8**, 15822 (2017).

82 Faesen, A. C. et al. Basis of catalytic assembly of the mitotic checkpoint complex. *Nature* **542**, 498-502 (2017).

83 Izawa, D. & Pines, J. The mitotic checkpoint complex binds a second CDC20 to inhibit active APC/C. *Nature* **517**, 631 (2014).

84 Di Fiore, B., Wurzenberger, C., Davey, N. E. & Pines, J. The mitotic checkpoint complex requires an evolutionary conserved cassette to bind and inhibit active APC/C. *Mol. Cell* **64**, 1144-1153 (2016).

85 Burton, J. L. & Solomon, M. J. D box and KEN box motifs in budding yeast Hsl1p are required for APC-mediated degradation and direct binding to Cdc20p and Cdh1p. *Genes Dev.* **15**, 2381-2395 (2001).
