# Supplementary material for: Genomic analysis finds no evidence of canonical eukaryotic DNA processing complexes in a free-living protist
Source: Nat Commun. 2021 Oct 14;12:6003. doi: 10.1038/s41467-021-26077-2 (PMC8516963; doi:10.1038/s41467-021-26077-2)
Supplement: Supplementary file 3 — Description of Additional Supplementary Files [file 41467_2021_26077_MOESM3_ESM.pdf]

Description of Additional Supplementary Files

Supplementary Data 1: BUSCO proteins found in Metamonada based on searches for 245 proteins present in at least one taxon.

Supplementary Data 2: DNA replication and repair orthologs in 18 diverse eukaryotic genomes.

Supplementary Data 3: Additional genomes queried during the searches for ORC, Cdc6 and Ndc80 proteins.

Supplementary Data 4: Spindle assembly, kinetochore and APC/C orthologs in 18 diverse eukaryotic genomes.

Supplementary Data 5: Lengths of Orc1-6, Cdc6 and Orc1/Cdc6-like proteins and domain architecture comparisons between metamonads and other eukaryotes.

Supplementary Data 6: Orc1, Cdc6 and Orc1/Cdc6-likeproteins.
