# Supplementary material for: Genomic analysis finds no evidence of canonical eukaryotic DNA processing complexes in a free-living protist
Source: Nat Commun. 2021 Oct 14;12:6003. doi: 10.1038/s41467-021-26077-2 (PMC8516963; doi:10.1038/s41467-021-26077-2)
Supplement: Supplementary file 10 — Reporting Summary [file 41467_2021_26077_MOESM10_ESM.pdf]

nature portfolio

Corresponding author(s): Dayana E. Salas-Leiva
Andrew J. Roger

Last updated by author(s): Sep 01, 2021

Reporting Summary

Nature Portfolio wishes to improve the reproducibility of the work that we publish. This form provides structure for consistency and transparency in reporting. For further information on Nature Portfolio policies, see our Editorial Policies and the Editorial Policy Checklist.

Statistics

For all statistical analyses, confirm that the following items are present in the figure legend, table legend, main text, or Methods section.

n/a	Confirmed	
☒	☐	The exact sample size (*n*) for each experimental group/condition, given as a discrete number and unit of measurement
☒	☐	A statement on whether measurements were taken from distinct samples or whether the same sample was measured repeatedly
☒	☐	The statistical test(s) used AND whether they are one- or two-sided<br>*Only common tests should be described solely by name; describe more complex techniques in the Methods section.*
☒	☐	A description of all covariates tested
☒	☐	A description of any assumptions or corrections, such as tests of normality and adjustment for multiple comparisons
☒	☐	A full description of the statistical parameters including central tendency (e.g. means) or other basic estimates (e.g. regression coefficient) AND variation (e.g. standard deviation) or associated estimates of uncertainty (e.g. confidence intervals)
☒	☐	For null hypothesis testing, the test statistic (e.g. *F*, *t*, *r*) with confidence intervals, effect sizes, degrees of freedom and *P* value noted<br>*Give P values as exact values whenever suitable.*
☒	☐	For Bayesian analysis, information on the choice of priors and Markov chain Monte Carlo settings
☒	☐	For hierarchical and complex designs, identification of the appropriate level for tests and full reporting of outcomes
☒	☐	Estimates of effect sizes (e.g. Cohen's *d*, Pearson's *r*), indicating how they were calculated

Our web collection on statistics for biologists contains articles on many of the points above.

Software and code

Policy information about availability of computer code

Data collection	All the software used was open source: Albacore v2.3.3, Porechop v0.2.3, and ABruijn v1.0 for base calling, long read trimming, and genome assembly, respectively. Polishing and error correction of the assembled genome was carried out with Nanopolish v0.10.1 and Unicycler v0.4.4, respectively.
Data analysis	All the software used was open source: Trimmomatic v0.39, Samtools v1.11 and mosdepth v0.2.5, Trinity v2.5.0, TransDecoder v5.5.0, RepeatModeler v1.0, RepeatMasker v4.0.7, Hisat v2.1.0, Bowtie v2.3.1, GenMarkET 4.38, Augustus v3.2.3, PASA v2.3.3, Merqury v1.3, BUSCO v3.0.2, BLAST suite v.2.7.1 (programs BLASTn, BLASTp and tBLASTn), exonerate v2.54.1, ploidyNGS v3.0, HMMER v3.1b2, MAFFT v7.310, IQ-TREE v1.6.5, ETE tools v3.1.1 and BMGE v1.0.

For manuscripts utilizing custom algorithms or software that are central to the research but not yet described in published literature, software must be made available to editors and reviewers. We strongly encourage code deposition in a community repository (e.g. GitHub). See the Nature Portfolio guidelines for submitting code & software for further information.

Data

Policy information about availability of data

All manuscripts must include a data availability statement. This statement should provide the following information, where applicable:

- Accession codes, unique identifiers, or web links for publicly available datasets
- A description of any restrictions on data availability
- For clinical datasets or third party data, please ensure that the statement adheres to our policy

Genome assembly is available at NCBI under BioProject PRJNA719540 and accession number JAHDYR000000000, and RNA-seq is available under accession SRR15678499. High resolution figures embedded in the Supplementary Information are available at https://doi.org/10.5061/dryad.wh70rxwnv

Field-specific reporting

Please select the one below that is the best fit for your research. If you are not sure, read the appropriate sections before making your selection.

☐ Life sciences ☐ Behavioural & social sciences ☒ Ecological, evolutionary & environmental sciences

For a reference copy of the document with all sections, see nature.com/documents/nr-reporting-summary-flat.pdf

Ecological, evolutionary & environmental sciences study design

All studies must disclose on these points even when the disclosure is negative.

Study description	We sequenced the genome of the protist Carpediemonas membranifera BICM and carried out a bioinformatics descriptive analysis that reconstructed protein complexes involved in its DNA replication, repair, and segregation.
Research sample	Genome sequencing was carried out for the C. membranifera strain BICM from littoral anoxic sediments in British Columbia (Canada) that was was isolated and reported by Kolisko et al 2010 (https://doi.org/10.1111/j.1462-2920.2010.02239.x)
Sampling strategy	Does not apply. DNA and RNA extraction was obtained by harvesting and pooling together all cultured cells from multiple cultures containing C. membranifera BICM. One single run of RNA-seq was obtained and used for assisting gene prediction.
Data collection	Sequencing of C. membranifera BICM strain was done with Illumina short paired-end and long MinION read technologies. The Illumina sequencing employed DNA from a monoxenic culture grown in 50 ml Falcon tubes in F/2 media enriched with the bacterium Shewanella frigidimarina as food. DNA was isolated from a total of two litres of culture using a salt extraction protocol followed by CsCl gradient centrifugation. RNA was also extracted from these cultures using TRIzol (Invitrogen, USA), following the manufacturer's instructions. For MinION sequencing, C. membranifera was grown in sterile filtered 50% natural sea water media with 3% LB with either Shewanella sp or Vibrio sp. isolate JH43 as food. Cell cultures were harvested by centrifugation at 500×g, 8 min, 20 ºC. The cells were resuspended in sterile-filtered spent growth media (SFSGM) and centrifuged again at 500×g, 8 min, 20 ºC. The cell pellets were resuspended in 1.5 mL SFSGM, layered on top of 9 mL Histopaque®-1077 (Sigma-Aldrich) and centrifuged at 2000×g, 20 min, 20 ºC. The protists were recovered from the media:Histopaque interface by pipetting, diluted in 10 volumes of SFSGM and centrifuged 500×g, 8 min, 20 ºC. High molecular weight DNA was extracted using MagAttract HMW DNA Kit (Qiagen, Cat No. 67563), purified with GenomicTip 20/G (Qiagen, Cat No. 10223) and resuspended in 5 mM Tris-HCl (pH 8.5).
Timing and spatial scale	Spacial scale: microbial cultures. Cells were collected during log and stationary phases
Data exclusions	No data were excluded from the analyses.
Reproducibility	Does not apply. This was not an experimental study but a descriptive analysis in which multiple cultures were grown and pooled together to obtain enough cells to yield enough extracted DNA to assemble a genome.
Randomization	Does not apply. This was not an experimental study but a bioinformatics descriptive research of a newly sequenced genome
Blinding	Does not apply. This was not an experimental study but a bioinformatics descriptive research of a newly sequenced genome

Did the study involve field work? ☐ Yes ☒ No

Reporting for specific materials, systems and methods

We require information from authors about some types of materials, experimental systems and methods used in many studies. Here, indicate whether each material, system or method listed is relevant to your study. If you are not sure if a list item applies to your research, read the appropriate section before selecting a response.

Materials & experimental systems

n/a	Involved in the study
☒	☐ Antibodies
☒	☐ Eukaryotic cell lines
☒	☐ Palaeontology and archaeology
☒	☐ Animals and other organisms
☒	☐ Human research participants
☒	☐ Clinical data
☒	☐ Dual use research of concern

Methods

n/a	Involved in the study
☒	☐ ChIP-seq
☒	☐ Flow cytometry
☒	☐ MRI-based neuroimaging
